# Contrasting population genetic structure in three aggregating groupers (Percoidei: Epinephelidae) in the Indo-West Pacific: the importance of reproductive mode

**DOI:** 10.1186/s12862-018-1284-0

**Published:** 2018-12-04

**Authors:** Ka Yan Ma, Lynne van Herwerden, Stephen J. Newman, Michael L. Berumen, John Howard Choat, Ka Hou Chu, Yvonne Sadovy de Mitcheson

**Affiliations:** 1 Simon F. S. Li Marine Science Laboratory, School of Life Sciences, The Chinese University of Hong Kong, Shatin, Hong Kong SAR, China; 2 College of Science and Engineering, James Cook University, Douglas, Townsville, QLD 4811 Australia; 3 Western Australian Fisheries and Marine Research Laboratories, Department of Primary Industries and Regional Development, Government of Western Australia, PO Box 20, North Beach, WA 6920 Australia; 4 Red Sea Research Center, Division of Biological and Environmental Sciences, King Abdullah University of Science and Technology, Thuwal, Saudi Arabia; 5 Swire Institute of Marine Science, School of Biological Sciences, University of Hong Kong, Pokfulam Road, Hong Kong SAR, China

**Keywords:** Phylogeography, Connectivity, Control region, Microsatellite, Pleistocene glaciation, Reproduction, Spawning aggregation

## Abstract

**Background:**

Understanding the factors shaping population genetic structure is important for evolutionary considerations as well as for management and conservation. While studies have revealed the importance of palaeogeographic changes in shaping phylogeographic patterns in multiple marine fauna, the role of reproductive behaviour is rarely considered in reef fishes. We investigated the population genetics of three commercially important aggregating grouper species in the Indo-West Pacific, namely the camouflage grouper *Epinephelus polyphekadion*, the squaretail coral grouper *Plectropomus areolatus*, and the common coral trout *P. leopardus,* with similar life histories but distinct spatio-temporal characteristics in their patterns of forming spawning aggregations.

**Results:**

By examining their mitochondrial control region and 9–11 microsatellite markers, we found an overarching influence of palaeogeographic events in the population structure of all species, with genetic breaks largely coinciding with major biogeographic barriers. The divergence time of major lineages in these species coincide with the Pleistocene glaciations. Higher connectivity is evident in *E. polyphekadion* and *P. areolatus* that assemble in larger numbers at fewer spawning aggregations and in distinctive offshore locations than in *P. leopardus* which has multiple small, shelf platform aggregations.

**Conclusions:**

While palaeogeographic events played an important role in shaping the population structure of the target species, the disparity in population connectivity detected may be partly attributable to differences in their reproductive behaviour, highlighting the need for more investigations on this characteristic and the need to consider reproductive mode in studies of connectivity and population genetics.

**Electronic supplementary material:**

The online version of this article (10.1186/s12862-018-1284-0) contains supplementary material, which is available to authorized users.

## Background

A primary objective of phylogeography is to identify the historical events that have shaped the current population genetic structure within species [1]. In the Indo-Pacific, the Pleistocene glaciations have left pronounced evolutionary footprints on a wide range of marine fauna. These glacial cycles resulted in significant reduction of coastal marine habitats and the emergence of land barriers, such as the Indo-Pacific Barrier (IPB) between the Indian and Pacific Oceans [2], and the Red Sea Barrier (RSB) at the shallow (137 m) and narrow (18 km) strait at Bab al Mandab, the sole connection between the Red Sea and the Indian Ocean [3]. The associated habitat loss [4, 5] and population isolation [6] led to dramatic population bottlenecks [5], which might have facilitated genetic differentiation among allopatric populations as genetic drift operates more efficiently in smaller populations. Moreover, environmental differences such as salinity or temperature may generate selective pressures that fast track divergence between isolated populations in different environments [7]. Nonetheless, although some reef fishes exhibit strong genetic partitioning at the IPB and RSB with very limited genetic exchange, such as damselfish [8], parrotfish [9]*,* angelfish and squirrelfish [10], some species, particularly those with high dispersal potential, are genetically homogeneous throughout the Indo-Pacific, such as unicornfishes [11, 12], and moray eels [13]. Hence, while phylogeographic patterns of organisms are often interpreted through past environmental disturbances, mediated by climate changes and geographic barriers, they may also be strongly influenced by species-specific traits [14]. The latter has not received much consideration.

The evolution of groupers (family Epinephelidae), a commercially important and speciose taxon of considerable biomass in reef ecosystems, was significantly impacted by environmental change associated with glacial cycles, as revealed by a recent historical biogeographic study [15]. These changes resulted in increased allopatric divergences across major biogeographic barriers (e.g. IPB and RSB) during the Pliocene and Pleistocene when sea levels dropped (5.3–0.01 million years ago (MYA)). However, little is known about the effect of Pleistocene climate change on intraspecific diversification of groupers, particularly whether or not sea level changes over the last four glacial periods has led to finer-scale population differentiation within biogeographic regions, as has been documented for other species of coral reef fishes such as the ocellaris clownfish [16] and the common coral trout [17]. Moreover, there are limited studies on whether co-distributed species that differ only in one or a few major trait(s) exhibit marked differences in their population genetic structure.

**Fig. 1:**
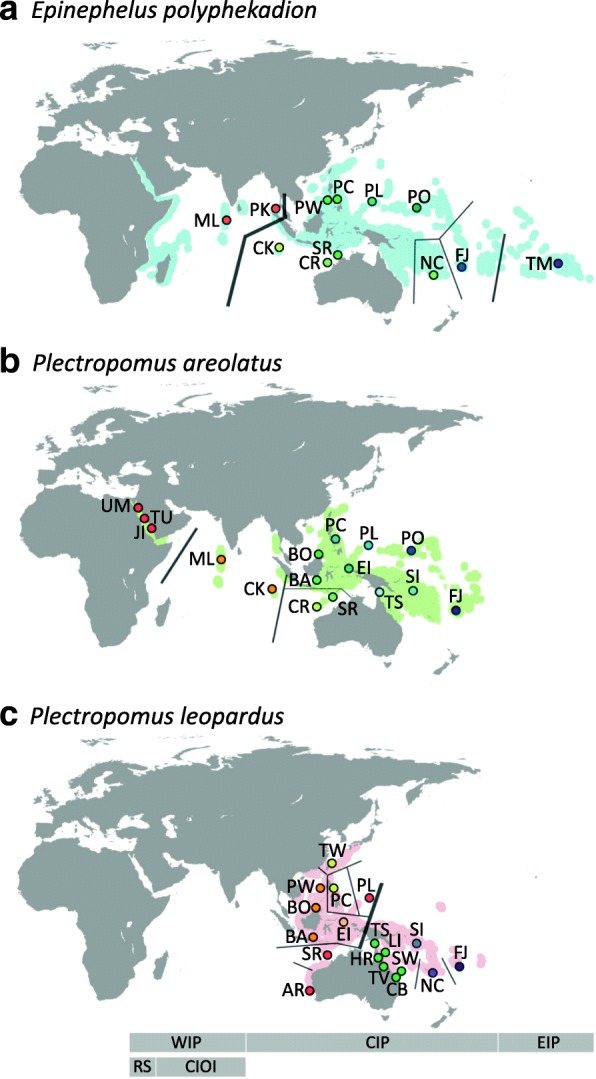
Maps showing distribution ranges and sampling sites of three target species. Colours of symbols correspond to the population genetic structure inferred by SAMOVA based on control region sequences from (**a**) *Epinephelus polyphekadion*, (**b**) *Plectropomus areolatus*, and (**c**) *P. leopardus*, while lines illustrate population genetic structure based on microsatellite datasets as deduced from SAMOVA analyses (see Additional file 1: Table S9 for detailed SAMOVA results). The legend beneath the panel of maps depicts longitudinal boundaries of marine ecoregions (following Spalding et al. 2007) included in this study: WIP: Western Indo-Pacific; RS: Red Sea; CIOI: Central Indian Ocean Islands; CIP: Central Indo-Pacific; EIP: Eastern Indo-Pacific. (see Figs. 2 and 3, and Additional file 1: Tables S2–4, A6–8 for code of sampling sites)

Here, we examined the population genetic structure of three common and commercially important groupers in the Indo-West Pacific: the camouflage grouper *Epinephelus polyphekadion* (Bleeker, 1849), the square-tail coral grouper *Plectropomus areolatus* (Rüppell, 1830), and the common coral trout, *P. leopardus* (Lacepède, 1802). These groupers are top predators on coral reefs that feed predominantly on fish [18] and hence have an important ecological role as part of the predator biomass that helps to shape the reef ecosystem. These piscivores are large (some exceeding 1 m in total length), roaming in shallow (usually < 50 m, but some occur at 300 m) tropical and subtropical waters throughout the Indo-Pacific and largely co-occur in the Pacific [19], although *P. leopardus* has a restricted distributional range relative to the other two species (Fig. 1). All of them are common enough throughout their distribution ranges to support fisheries [19]. Their pelagic larval durations (PLDs) are thought to range from 3 to 6 weeks [20, 21] and home ranges in non-reproductive periods are generally less than 5 km^2^ [22]. The three species all aggregate to spawn whereby gamete release takes place over a relatively short period (a few days) during a few to multiple spawning months. Individuals can display high spawning site fidelity, as determined from tagging studies [23, 24]. However, they differ substantially in the degree to which their spawning aggregation is spatially, numerically and temporally concentrated (see paragraphs below).

The formation of spawning aggregations is a reproductive strategy exhibited by many fish families, including coral reef species such as groupers, snappers, jacks (trevallies), surgeonfishes, damselfishes and parrotfishes [25–29]. Fish spawning aggregations (FSA) of groupers that exhibit this reproductive mode are typically brief, spatially restricted, gatherings of tens to tens of thousands of reproductive individuals drawn from a wider catchment area, which can include fish that have travelled tens to hundreds of kilometres to reach the aggregation site [30]. This is in marked contrast to many reef fishes which spawn within or close to their home range over long periods of time each year [31, 32]. FSAs may couple with short-term oceanographic conditions such as slack currents which enhance fertilization success or they may coincide with rapid currents that could strongly influence either larval retention or dispersal [33–35]. Some aggregations may occur on the reef platform, others only at the shelf edge exposed to extreme oceanic conditions. Such extreme temporal and spatial concentrations (point sources and locations) of gamete release during annual spawning events may confer significant impacts on a species’ population connectivity, but this factor has rarely been considered.

Among our target species, *P. leopardus* forms multiple small (a few hundreds of individuals) aggregations for 1–2 months each year and can migrate up to 10 km between spawning sites and home ranges [24], while *P. areolatus* forms relatively fewer, yet much larger (up to thousands of individuals), aggregations for multiple months (up to 8 months each year, although fewer months in some locations), with spawning migrations of up to 30 km [22, 36]. By contrast, *E. polyphekadion* forms few, large (thousands of individuals), spawning aggregations for one or sometimes two months a year, with adults known to travel up to 40 km to spawning sites from their home reefs (K. Rhodes – personal communication). The latter two species aggregate at shelf edge/drop-off areas, often associated with outer reef channels and close to offshore waters, for a few days around the full or new moon (depending on location) [37], whereas *P. leopardus* characteristically spawns on the shelf platform on the full moon in Australia [38]. The distinctive temporal and spatial differences in the spawning aggregations of the three species may have differentially impacted their phylogeographic patterns. Specifically, a more concentrated (i.e. in time and space) release of gametes and/or more frequent spawning at offshore sites, as evident in *P. areolatus*, may have evolved to favour long distance larval dispersal, which may have weakened the phylogeographic signal in this species.

This study aims to test whether the phylogeographic patterns of the three grouper species (1) are shaped by past environmental changes, and (2) whether disparity in population genetic structure among the species, if any, could be attributable to differences in their reproductive traits. In this regard, we would predict *P. areolatus* to exhibit the lowest level of genetic population structure across its geographic range because of the concentrated release of gametes and more frequent spawning that could have evolved to favour long distance dispersal. By contrast, we expected *P. leopardus* to have the strongest genetic population structure across its range, being a poor disperser relative to the other two species, due to smaller and spatially scattered aggregations that are not close to offshore waters. To test these hypotheses, we conducted an extensive genetic examination over the entire range of the three species by using the mitochondrial control region and nuclear microsatellite markers. Moreover, since our target species are among the most commercially important reef fish in the Indo-Pacific, and some are already threatened by overfishing, connectivity information would be valuable to improve their management and conservation. We evaluate our findings against comparable population genetics work in other groupers and consider possible management and conservation implications.

## Methods

### Sample collection and DNA extraction

Fin clips or muscle tissues from the three species were obtained from 28 locations throughout the Indo-Pacific (Fig. 1). Sampling was undertaken by selective spearing or line fishing, or were acquired from local fish markets. All tissues were preserved immediately in 90% ethanol or NetStar solution. Total DNA was extracted using a QIAamp Tissue Kit (QIAGEN) or 5% Chelex solution [39].

### Mitochondrial DNA (mtDNA) analyses

A 580-base pair (bp) fragment of mtDNA control region was amplified using primers developed for this study (see Additional file 1: Table S1). Polymerase chain reaction (PCR) mixtures contained 1–5 μl of template DNA, 1X PCR reaction buffer, consisting of 3 mM MgCl_2_, 200 μM dNTPs, 200 nM of each primer, 1.5 U of Taq polymerase (Amersham) and ddH_2_O to a total volume of 50 μl. The thermal cycle consisted of an initial denaturation at 94 °C for 3 min followed by 38 cycles at 94 °C for 30 s, 54 °C for 50 s, 72 °C for 1 min 30 s, with a final extension at 72 °C for 10 min. PCR products were purified using Millipore Montage PCR96 Cleanup Kit, following manufacturer’s instructions. Sequencing was performed in the forward direction using an Applied Biosystems 3100 sequencer and followed standard cycle sequencing protocols (BGI, China). Sequence data were edited using MEGA 5.0 [40] and aligned using MUSCLE [41] with default settings, as implemented in MEGA 5.0 and adjusted manually. The haplotype diversity (*h*) and nucleotide diversity (π) [42] were assessed using Arlequin 3.5 [43]. Neutrality tests (Tajima’s D [44], and Fu’s F_S_ [45]) were conducted in Arlequin with 1000 permutations.

Population genetic structure was examined by three means: (1) SAMOVA 2.0 [46] was used to analyse the spatial genetic structure with 1000 permutations and the number of initial conditions was set to 100. The maximum proportion of missing data was set as 0.5%. If the number of individuals analysed at a site (e.g. Bali and Borneo) was less than 16, the site(s) would either be removed or grouped with nearby sites (as noted in the footnote to Additional file 1: Tables S2–4). We tested the number (*K*) of groups of populations from 2 to the number of populations minus 1; the *K* value with the highest and significant *F*_*CT*_ was regarded as the best representation of the number of distinctive groups for that dataset. SAMOVA was run with and without geographic information, which gave consistent results. To reduce errors in significance calculations due to missing data, selected grouping schemes were subjected to locus-by-locus Analysis of Molecular Variance (AMOVA) in Arelquin 3.5; (2) Pairwise Φ_ST_ statistics were computed using Arlequin with 99,999 permutations; and (3) The evolutionary relationships among haplotypes and genetic structure were evaluated and visualized by a minimum spanning network (MSN) analysed using Arlequin and drawn using Gelphi [47] and Adobe Illustrator.

Based on the average nucleotide substitution rate of the control region of 10% Myr^− 1^ between lineages [48], the divergence time among genetic clades was approximately estimated.

### Microsatellite analyses

Between nine and eleven microsatellite loci were analysed in the three species examined (Additional file 1: Table S5). All PCR mixes contained 1.25–1.5 μl of template DNA, 1X KAPA2G Buffer A, 3 mM MgCl_2_, 150 μM dNTPs, 60–240 nM of each fluorescent-labelled (FAM, VIC, NED or PET) forward primer, 60–240 nM of each reverse primer, 4% Bovine Serum Albumin (BSA) and 1.5 U KAPA2G Fast DNA polymerase in a total volume of 6.25 μl. A touch-down thermal programme was used: initial denaturation at 95 °C for 2 min, followed by 30 cycles of denaturation at 95 °C for 10 s, 12 s at annealing temperature stepping down 0.2 °C per cycle from 62 °C to 56 °C and an extension at 68 °C for 3 s. Subsequently, another 10 cycles of denaturation at 95 °C for 10 s, 12 s annealing at 56 °C and extension at 68 °C for 3 s, were run. PCR products were genotyped using an Applied Biosystems 3100 DNA Analyzer along with GeneScan LIZ-500 (Applied Biosystems) as internal size standard. Allele sizes were analysed using GENEMARKER 2.4.0 (Softgenetics, LLC). MICROCHECKER 2.2.3 [49] was used to test for null alleles, large allele dropout and scoring errors. We used FreeNA [50] to test for the influence of null alleles by estimating *F*_*ST*_ before and after ENA (excluding null alleles). Correction for null alleles and bootstrapping (10,000 pseudoreplicates) was used to determine significance levels. The observed and expected heterozygosities (H_o_ and H_e_) were estimated for each locus using GenALEx 6.5 [51]. Significant deviations from Hardy-Weinberg equilibrium (HWE) at each locus and linkage disequilibrium (LD) between all loci [52] were tested by running 10,000 Markov chain iterations in the online version of GENEPOP 4.0 [53].

Genotypic population structure was assessed using four approaches: (1) GenALEx was used to estimate pairwise *F*-statistics; (2) SAMOVA was used to analyse the spatial genetic structure as previously described for mtDNA. The maximum proportion of missing data was set at 5%; (3) A discriminant analysis of principal components (DAPC) [54] was employed to produce scatterplots of discriminant functions derived from the spatial distribution of microsatellite genotypes. We retained the number of principal components (PCs) after which little information was gained by adding PCs, and chose the optimal number of groups according to Bayesian information criterion (BIC); and (4) Population genetic structure was examined using a Bayesian approach implemented in STRUCTURE 2.3.3 [55] under an admixture model with correlated allele frequencies and sampling locations as priors. Runs of 1 million steps and 20% burn-in for ten replicates of each value of K (from K = 1 to K = 12) were performed. STRUCTURE HARVESTER web 0.6.93 [56] was used to determine the best K for the dataset using the method of Evanno, Regnaut & Goudet [57]. We then divided the data by major groups to find hierarchical structure [57, 58].

## Results

### Molecular characteristics

This study analysed 723-bp, 580-bp and 556-bp segments of control region from 270 *E. polyphekadion*, 341 *P. areolatus* and 340 *P. leopardus*, respectively (Additional file 1: Tables S2–4, GenBank accession numbers KM656497-KM656787, MH853841-MH854500). Overall haplotype diversity (h) was high, ranging from 0.920 ± 0.01 in *P. areolatus* to 0.993 ± 0.002 in *E. polyphekadion,* while nucleotide diversity (π) ranged from 0.025 ± 0.013 in *P. areolatus* to 0.131 ± 0.063 in *P. leopardus.*

A total of 261, 397 and 365 individuals of *E. polyphekadion*, *P. areolatus* and *P. leopardus*, respectively, were genotyped for the microsatellite loci (Additional file 1: Tables S6–8). Large allele dropout and scoring errors were not detected in our datasets, but null alleles were present in 4 to 8 loci in 1 to 7 populations (Additional file 1: Table S5). Null allele frequency estimated by FreeNA ranged from 0 to 0.387 (mean 0.05) in *E. polyphekadion*, from 0 to 0.315 (mean 0.02) in *P. areolatus*, and from 0 to 0.317 (mean 0.076) in *P. leopardus* (data not shown). No significant disparity in global *F*_*ST*_ values computed with and without ENA correction was found (Additional file 1: Table S5). No pair of loci exhibited significant linkage disequilibrium in more than two populations (with α = 0.01, Additional file 1: Table S16). Therefore, we used all microsatellite loci in subsequent analyses. Locus-specific H_o_ and H_e_ ranged from 0.000 to 0.938 and from 0.000 to 0.909 in *E. polyphekadion*, from 0.000 to 1.000 and from 0.000 to 0.898 in *P. areolatus*, from 0.054 to 0.929 and from 0.053 to 0.913 in *P. leopardus* (Additional file 1: Tables S6–8). Significant deviation from HWE (Benjamini-Hochberg adjusted *P* < 0.01) was detected in 15/99, 8/126 and 28/108 cases *E. polyphekadion, P. areolatus and P. leopardus*, respectively, most of which were due to heterozygote deficiency (Additional file 1: Tables S6–8).

### Population genetic structure

#### *Epinephelus polyphekadion*

**Fig. 2:**
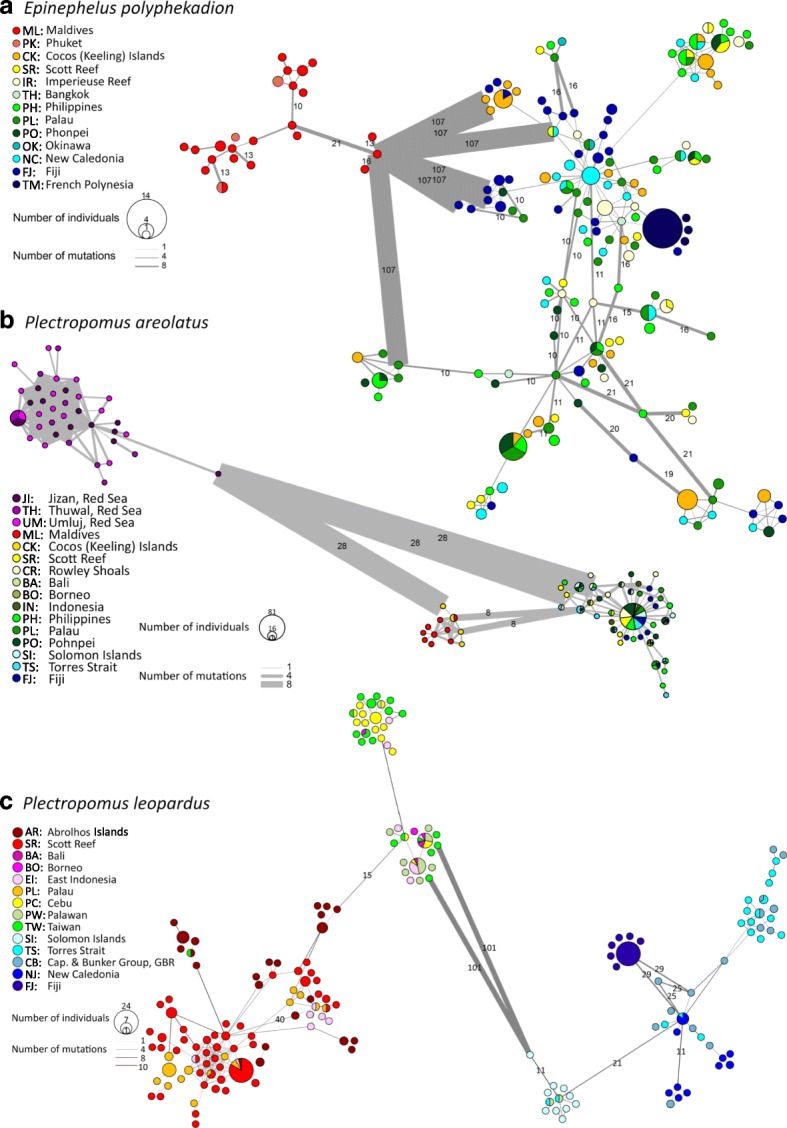
Control region haplotype MSN of (**a**) *Epinephelus polyphekadion*, (**b**) *Plectropomus areolatus*, and (**c**) *P. leopardus.* Each circle denotes one haplotype, with size proportional to number of individuals and colour representing sampling locality. The thickness of branches is proportional to the number of mutational steps between haplotypes, with numbers on branches indicating mutational steps ≥10

**Fig. 3:**
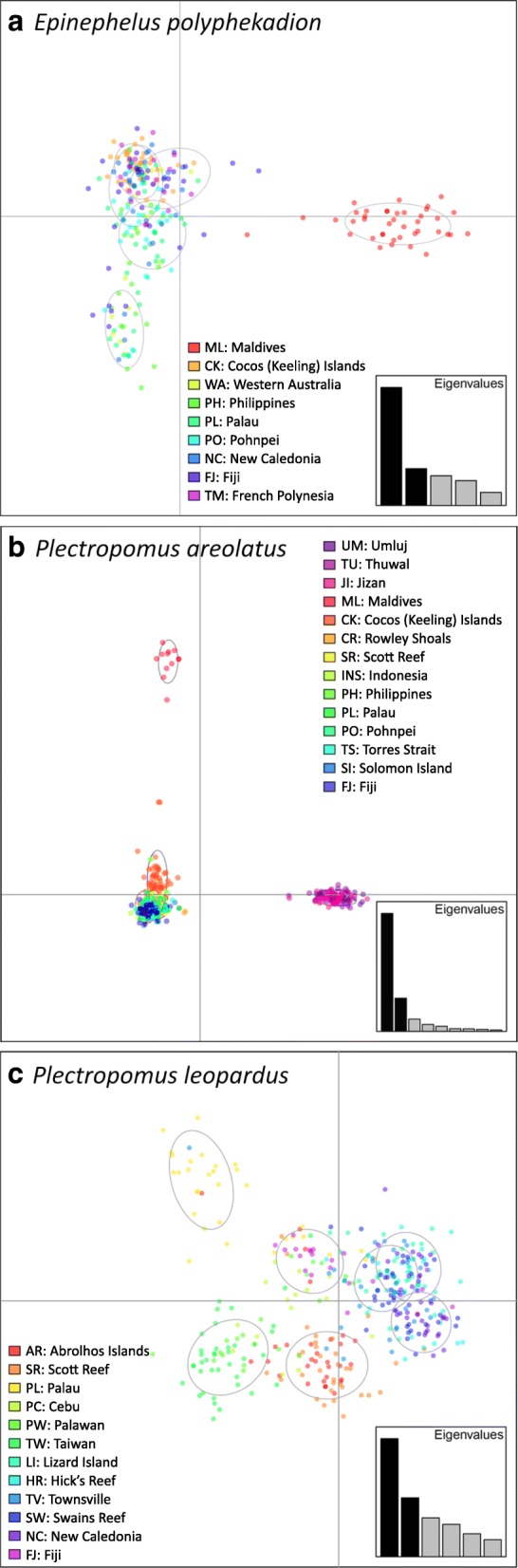
DAPC scatter plots of (**a**) *Epinephelus polyphekadion*, (**b**) *Plectropomus areolatus*, and (**c**) *P. leopardus*. c 1: Tables S6–8 for codes of sampling sites

**Fig. 4:**
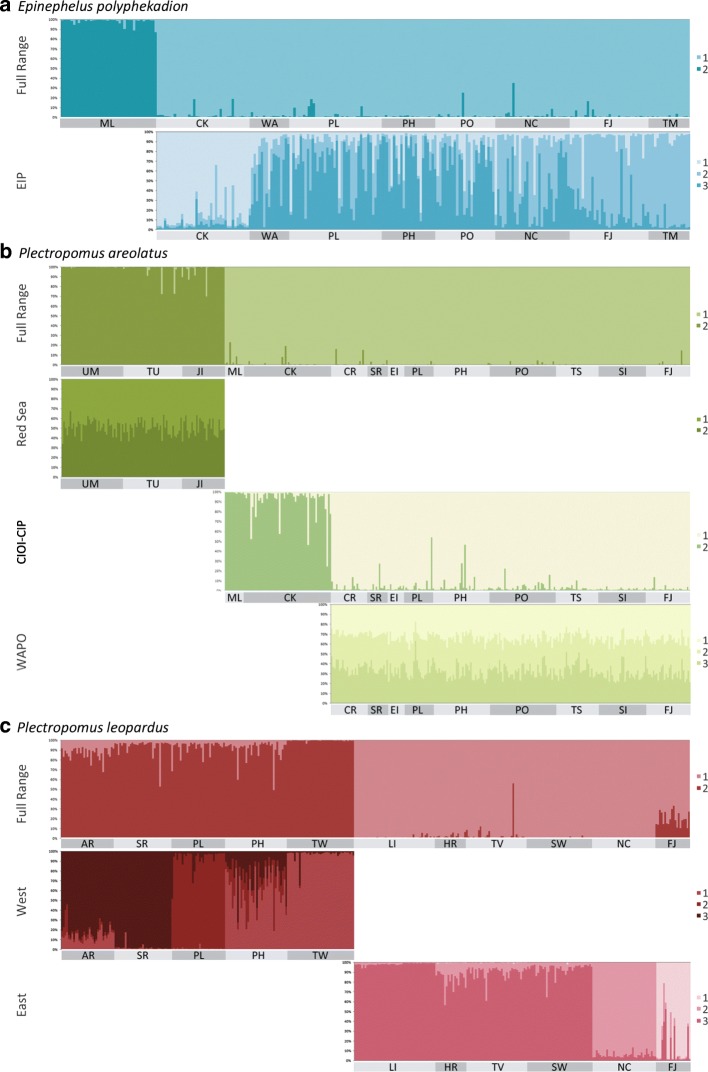
STRUCTURE assignment plots based on microsatellite loci for (**a**) *Epinephelus polyphekadion*, (**b**) *Plectropomus areolatus*, and (**c**) *P. leopardus*. See Additional file 1: Tables S6–8 for codes of sampling sites

The MSN of the control region (Fig. 2a) revealed two distinct groups separated by 107 mutational steps: one in the Western Indo-Pacific (WIP); the other in the Central Indo-Pacific (CIP) combined with the Eastern Indo-Pacific (EIP) (following Spalding et al. [59]). The average genetic distance amongst these two groups was 14% (± 1.2%), and the molecular clock suggested a divergence time of 1.41 ± 0.12 MYA (Table 1). Fine-scale population genetic structure within groups was not evident in the MSN, with the exception that all the peripheral French Polynesian haplotypes were closely related to each other, unlike any other populations. Results from microsatellite DAPC analyses were consistent with the mtDNA data, with the Maldives partitioned substantially along the first principal component, and the others only partitioned along the second principal component, which accounted for about a third of the total genetic variation (Fig. 3a). A separate DAPC analysis of the CIP populations (i.e. excluding the Maldives data) revealed five clusters, one containing mostly the Cocos (Keeling) Islands (CK) samples, two containing most of the Western Australian, Philippines, Palau and Pohnpei samples, and the remaining two containing mostly the New Caledonia, Fiji and French Polynesia samples (Additional file 1: Figure S1). Consistent with this, STRUCTURE analysis of the microsatellite data identified a WIP and a CIP-EIP group (Fig. 4a). A further STRUCTURE assignment analysis within the latter group revealed finer scale population genetic structure as noted before, with almost all CK samples assigned to group 1, most samples from Western Australia, Palau, Philippines and Pohnpei assigned to group 2, and an increasing trend of assignments to group 3 from New Caledonia eastward to French Polynesia (Fig. 4a).

**Table 1:** Inter-lineage genetic distances and divergence times

	Mean between group p-distance (mean ± stdev^a^)	Divergence time
*E. polyphekadion*		
WIP vs CIP-EIP	0.141 ± 0.012	1.41 ± 0.12 MYA
*P. areolatus*		
WAPO vs Maldives + CK	0.016 ± 0.006	0.16 ± 0.06 MYA
Red Sea vs WAPO + Maldives + CK	0.062 ± 0.012	0.62 ± 0.12 MYA
*P. leopardus*		
East vs West	0.169 ± 0.018	1.69 ± 0.18 MYA

^a^Stdev: Standard deviations were estimated by 100 bootstrap replicates

SAMOVA computed the highest *F*_*CT*_ when K = 2 in both the control region (standard *F*_*CT*_ = 0.852, *P* = 0.105, locus by locus *F*_*CT*_ = 0.852, *P* < 0.0001) and in microsatellites (standard *F*_*CT*_ = 0.265, *P* = 0.129, locus by locus *F*_*CT*_ = 0.197, *P* < 0.0001, Additional file 1: Table S9), suggesting that *E. polyphekadion* is comprised of two distinctive groups: (1) WIP, including the Maldives and Phuket, and (2) the CIP-EIP, including samples from CK to French Polynesia (see Fig. 1A). SAMOVA within the latter group revealed considerable genetic partitioning between CIP and EIP in the control region (standard *F*_*CT*_ = 0.219, *P* = 0.123, locus by locus *F*_*CT*_ = 0.218, *P* < 0.0001) and in microsatellites (standard *F*_*CT*_ = 0.066, *P* = 0.129, locus by locus *F*_*CT*_ = 0.038, *P* < 0.05, Additional file 1: Table S9). SAMOVA of the CIP revealed very weak, but significant population structuring (control region standard *F*_*CT*_ = 0.046, *P* < 0.01, locus by locus *F*_*CT*_ = 0.046, P < 0.0001; microsatellite standard *F*_*CT*_ = 0.051, *P* < 0.05, locus by locus *F*_*CT*_ = 0.033, *P* < 0.01, Additional file 1: Table S9). *F*-statistics indicated the existence of a strong genetic break located west of CK (Φ_ST_ > 0.842 in mtDNA and *F*_*ST*_ > 0.127 in microsatellites, with all *P* < 0.001, Additional file 1: Tables S10, 11). Another lower magnitude genetic break was identified east of Fiji (Φ_ST_ > 0.290 and *F*_*ST*_ > 0.033) in mtDNA and microsatellites with all *P* < 0.001). Population differentiation within the CIP was generally weak (Φ_ST_ - 0.108 to 0.132, *P* < 0.00001) in four of 36 cases and *F*_*ST*_ - 0.035 to 0.092, *P* < 0.00001 in six of 15 cases), but CK was a genetically distinct population from all other populations based on microsatellites (*F*_*ST*_ > 0.036, all *P* < 0.001). We detected no sign of recent population expansion in the WIP group (Tajima’s D -1.173, *P* = 0.105; Fu’s F_S_ -5.045, *P* = 0.038), while in the CIP-EIP group, Fu’s F_S_, which is more sensitive to sudden demographic expansion than Tajima’s D, was significantly negative (- 23.705, *P* < 0.01), while Tajima’s D was not significant (- 1.618, *P* = 0.017).

#### *Plectropomus areolatus*

The MSN of the control region (Fig. 2b) partitioned *P. areolatus* into three groups: Red Sea (RS), Maldives-CK, and the Western Australia–Pacific Ocean (WAPO), with the WAPO population genetically less distinct from the Maldives-CK population (separated by eight mutational steps) than from the RS clade (separated by 28 mutational steps). The average genetic distance amongst the WAPO and Maldives-CK group was 1.6% (± 0.6%), and amongst the RS and the rest was 6.2% (± 1.2%) and the molecular clock estimates of divergence time were about 0.16 ± 0.06 MYA and 0.62 ± 0.12 MYA, respectively (Table 1). Fine-scale population genetic structure within both the RS and the WAPO groups was not evident. Indeed, a dominant haplotype was shared in each geographic clade, by all samples. In the Maldives-CK group, however, the two sites mostly harboured private haplotypes and shared a single haplotype (Fig. 2b). DAPC analyses of the microsatellites suggested 10 clusters, but the scatter plot depicted significant dissimilarity only among three cluster-groups corresponding to the RS, Central Indian Ocean Islands (CIOI sensu Spalding et al. 2007, i.e. Maldives), and CIP samples, with the CK cluster showing some distinction from the remaining CIP samples along the second principal component only, accounting for ~ 25% of the genetic variation captured by the first principal component (Fig. 3b). DAPC analysis for the RS populations found no fine-scale population structuring among sites (Additional file 1: Figure S2). By contrast, DAPC analysis within Western and Central Indo-Pacific populations found distinction among the Maldives, CK and the WAPO populations, while a further DAPC analysis of the WAPO populations revealed subtle differences between the Western Australian populations and the others (Additional file 1: Figure S4). Similarly, the best-fitting K was 2 using STRUCTURE for the entire microsatellite dataset, partitioning it into a RS and a CIOI-CIP group (Fig. 4b), but an additional STRUCTURE assignment analysis of the CIOI-CIP group alone identified two groups corresponding to Maldives-CK and WAPO populations. No further partitioning was detected in STRUCTURE analyses of the RS and WAPO datasets.

The optimal grouping inferred by SAMOVA with the highest *F*_*CT*_ was congruent amongst both datasets in partitioning *P. areolatus* into three clusters, largely corresponding to the aforementioned pattern (control region standard and locus by locus *F*_*CT*_ = 0.898; microsatellite standard *F*_*CT*_ = 0.292, locus by locus *F*_*CT*_ = 0.290), all with *P* < 0.0001 (Additional file 1: Table S9). Similar to *E. polypekadion*, only very weak population structure was revealed within WAPO (control region standard and locus by locus *F*_*CT*_ = 0.045, *P* < 0.05; microsatellite standard and locus by locus *F*_*CT*_ = 0.043, *P* < 0.05, Additional file 1: Table S9). However, CK changed group membership between the two datasets. Specifically, CK grouped with either the Maldives in CIOI (control region, Φ_ST_ > 0.750, *P* < 0.001) or the WAPO region (microsatellites, *F*_*ST*_ > 0.228, *P* < 0.001). Both datasets identified the genetic break between the RS and Maldives (Additional file 1: Table S9). Similarly, *F*-statistics (Additional file 1: Tables S12, 13) revealed that the RS population was strongly isolated (control region pairwise Φ_ST_ > 0.926, microsatellite pairwise *F*_*ST*_ > 0.184, all *P* < 0.001) and that the CK population was genetically distinct from all others (control region pairwise Φ_ST_ > 0.161, *P* < 0.001, microsatellite pairwise *F*_*ST*_ > 0.049, all *P* < 0.001). Population differentiation within the WAPO was generally low and insignificant (Φ_ST_ - 0.922 to 0.146), with significant Φ_ST_ (*P* < 0.00001) only between Fiji and Rowley Shoals, based on the control region (Additional file 1: Table S12). However, microsatellite data suggested significant, albeit subtle differentiation (*F*_*ST*_ 0.002 to 0.075), specifically between West Australian sites and the rest, with *P* < 0.00001 in 22 of 36 comparisons (Additional file 1: Table S13). Likewise, within the RS population, there was no significant differentiation between locations for either dataset (Additional file 1: Tables S12, 13). All three groups showed signs of recent demographic expansion with their significantly negative Tajima’s D and Fu’s FS: WAPO Tajima’s D -2.033, *P* < 0.01; Fu’s F_S_ -26.984, *P* < 0.01; Maldives-CK: Tajima’s D -1.507, *P* < 0.05; Fu’s F_S_ -7.311, *P* < 0.01; RS: Tajima’s D -2.622, *P* < 0.01, Fu’s F_S_ -27.606, *P* < 0.01).

#### *Plectropomus leopardus*

The control region MSN (Fig. 2c) revealed two major groups that were separated by 101 mutational steps partitioned along Sahul Shelf. We herein termed the meta-population east of this break (from Torres Strait to Fiji) the Eastern population, and the meta-population west of it (from Western Australia to East Indonesia) the Western population. The average genetic distance amongst the two groups was 16.9% (± 1.8%), and the molecular clock suggested a divergence time of about 1.69 ± 0.18 MYA (Table 1). The Western population consisted of two groups separated by 15 mutational steps. One group contained all the Western Australian and Palauan *P. leopardus*, as well as some from East Indonesia and a Taiwanese individual. The other group was divided into two subgroups, ten mutational steps apart. One subgroup harboured mostly individuals from Taiwan and Cebu (central Philippines), while individuals from Palawan (west Philippines) and Indonesia dominated the other subgroup. The Eastern population contained three major groups: one group consisted mostly of Solomon Islands individuals, one harboured exclusively Fijian *P. leopardus*, and the remaining group contained individuals from the Torres Strait, Great Barrier Reef and New Caledonia. The DAPC analysis of microsatellites (Fig. 3c) also revealed an East-West division in this species, but the subpopulations were more mixed than revealed by the control region MSN. A further DAPC analysis within the Western population revealed four clusters, with one cluster containing almost exclusively all the Palauan samples, one containing predominantly Taiwanese samples, one containing mostly the Western Australian samples, and one containing mostly Philippine samples (Additional file 1: Figure S5). The DAPC analysis of the Eastern population identified six clusters, and the scatter plot showed strong genetic division between Fiji and the remaining populations (Additional file 1: Figure S6). Population assignment by STRUCTURE also identified two distinct *P. leopardus* clusters - an Eastern and a Western group, each of which could be further divided into three subgroups (Fig. 4c). The Western population contained three subgroups corresponding to Western Australian, Palauan, and Philippine-Taiwanese populations. The Eastern population contained three subgroups corresponding to a Great Barrier Reef, a New Caledonian and a Fijian population.

The *K* value for *P. leopardus* estimated by SAMOVA for the control region was nine (standard *F*_*CT*_ = 0.879, locus by locus *F*_*CT*_ = 0.795, all *P* < 0.0001) and almost all sites were partitioned, except Torres Strait grouped with the Capricorn Bunker Reefs, Cebu grouped with Taiwan, and Scott Reef grouped with Palau (Additional file 1: Table S9). In the microsatellite dataset, *K* was ten (standard *F*_*CT*_ = 0.250, *P* = 0.105, locus by locus *F*_*CT*_ = 0.133, *P* < 0.0001), with almost all sites being partitioned as before, except that three northern Great Barrier Reef sites (Lizard Island, Hick’s Reef, and Townsville) formed a single group (Additional file 1: Table S9). The strong population genetic structure was also supported by the fact that > 80% of control region pairwise Φ_ST_ and > 96% of microsatellite pairwise *F*_*ST*_ values were high (averaged 0.696 for Φ_ST_ and 0.116 for *F*_*ST*_) and were statistically significant (*P* < 0.001) (Additional file 1: Tables S14, 15). The *F*-statistics revealed a very strong genetic break between East Indonesia and Torres Strait (control region) and between Palau and the Great Barrier Reef (microsatellites), as pairwise *F*-statistics between sites on opposite side of this break were higher (mean Φ_ST_ = 0.935; mean *F*_*ST*_ = 0.102) than those on the same side (mean Φ_ST_ = 0.463; mean *F*_*ST*_ = 0.057). Both Tajima’s D (- 0.228, *P* = 0.466) and Fu’s F_S_ (- 10.640, *P* = 0.030) were negative but not significant in the Eastern group, while Fu’s F_S_ was significantly negative in the Western group (- 23.690, *P* < 0.01), Tajima’s D was not (- 0.782, *P* = 0.234).

## Discussion

### Influence of past environmental changes and geographic factors

This study identifies phylogeographic signatures of Pleistocene glacial cycles in all three species, with genetic breaks generally coinciding with major biogeographic boundaries - the most prominent one being the IPB (see Fig. 1). As shallow reef inhabitants, groupers could have experienced marked population bottlenecks due to coastal habitat loss associated with marine regression during Pleistocene glaciation [4, 5, 60], followed by recent demographic expansion, as suggested by the significantly negative Fu’s F_S_ in many of the grouper lineages from this study. The emergence of multiple land barriers including the IPB at the Sahul and Sunda Shelves [2] was one of the most significant barriers that impacted multiple fauna and could have physically segregated the grouper populations in CIOI and Indo-Polynesia (IP) [61], leading to genetic differentiation and/or speciation amongst these biogeographic compartments. Such patterns have frequently been observed in marine fauna, such as parrotfishes [9, 62], gobies [63], angelfish [64], gastropods [65], prawns [66] and multiple reef fishes [10, 67]. The notion that Pleistocene glaciation caused the genetic differentiation across IPB in groupers is supported in *E. polyphekadion* and *P. areolatus* based on the mid-Pleistocene divergence estimated by conventional molecular clock methods (1.41–0.16 MYA). Moreover, the IPB appears to present a dispersal boundary for *P. leopardus* such that the barrier might have resulted in the divergence of *P. leopardus* from its sister species *P. pessuliferus* (the roving coral grouper), which largely inhabited the west to central Indian Ocean, in the Pleistocene [15].

At the western periphery of the Indo-Pacific, the RSB exerted a profound effect on *P. areolatus*, causing the divergence of the RS lineage at about 0.63 MYA (mid-Pleistocene) based on the conventional molecular clock. Previous comparative phylogeographic work that focused on examining this barrier found genetic differentiation between the RS and Western Indian Ocean populations in five out of seven reef fish species that exhibit a wide spectrum of biological traits [10]. Similar genetic partitioning across the RSB was also detected in damselfish [68], crab [69], and starfish [70], but not in lionfish [71]. The RS was repeatedly isolated during the Pleistocene glacial cycle owing to the marked sea level drop – either through emergence of a land barrier or reduction of water flow associated with increased salinity and temperature [72, 73]. Such physical isolation and the strong selection pressure associated with the divergent environmental conditions at the peripheral habitats of a species’ distribution range are likely to have contributed to the genetic diversification observed in *P. areolatus* (this study) and in other fauna [74].

Intriguingly, despite the marine transgression that allowed for secondary contact of previously isolated populations, as evidenced in the peacock grouper (*Cephalopholis argus* [75]), we observed no contemporary connectivity across the IPB in either *E. polyphekadion* or *P. areolatus*. One likely explanation is that the extensive separation (> 2000 km) of suitable coral habitats and the relative absence of stepping stones in the deep ocean (> 200 m) for dispersal between the WIOI and IP, and between RS and WIOI (see Fig. 1) present a challenge to dispersal of these groupers even without land barriers [76]. Habitat discontinuity can result in significant population differentiation over short distances, even for marine fish with a pelagic larval phase, such as the corkwing wrasse [77] and triplefin blenny [78]. A combination of ecological, biological and environmental factors, rather than oceanography *per se*, may be more important in determining population genetic structure across major biogeographic barriers for the grouper species examined here.

At the eastern boundary of the species’ range (Tuamotus of French Polynesia), we detected subtle but significant isolation of the French Polynesian *E. polyphekadion*, which has lower genetic diversity (in both mitochondrial and microsatellite loci) than other populations. Multiple factors, including geographic isolation, oceanographic currents that influence gene flow, and selection pressure in distinct environmental conditions, may play a role in creating the genetic differentiation in this marginal population of *E. polyphekadion* [79, 80]. In fact, such patterns of extensive population connectivity across the wide range of the WIP with isolation at the eastern margin of the Pacific range is frequently detected in reef fishes, such as the peacock grouper [75], the brown surgeonfish [80], the bluestriped snapper [79], and the flame angelfish [81].

Our results revealed multiple genetically differentiated populations of *P. leopardus* in the WIP, with the deepest divergence at the Sahul Shelf (Fig. 1c). While the other populations were likely isolated by regional oceanographic features [17], the divergence of the East and West populations of *P. leopardus* was estimated at about 1.69 MYA (early Pleistocene), suggesting the possible contribution of the Pleistocene glaciation that resulted in the recurrent emergence of the Sahul shelf land bridge. The Sahul shelf land bridge has been documented as a barrier associated with genetic differentiation for several other tropical marine species, from starfish [82] to sea snakes [83]. Though the land bridge has been submerged for the past 9000 years, the region remained uninhabited by *P. leopardus* (see Fig. 1), and thus, given the lower dispersal potential of this species (see discussion below), the two meta-populations may maintain low connectivity and genetic differentiation. Another possible explanation for the pattern observed is that each isolated region has developed region-specific traits, though this notion remains speculative until the ecology of *P. leopardus* across its range is studied in more detail. Theoretically, region-specific traits may prevent realized dispersal during transgressions by selection against immigrants from other environments, as documented for Atlantic herring using genomic approaches [7]. This was also demonstrated in the *Dascyllus trimaculatus* (Rüppell, 1829) threespot dascyllus species complex, where historical allopatry developed during periods of isolation and subsequent ecological factors facilitated divergence and speciation of these species [8].

Notably, the affinity of the CK population, which is located at the WIP-CIP boundary, is different between *E. polyphekadion* and *P. areolatus*. In *E. polyphekadion*, the CK population groups with the IP population in all analyses (mitochondrial and microsatellite data). However, in *P. areolatus*, the CK population has a close affinity with the Maldives population in the WIOI, based on the mitochondrial control region, but it is more closely related, albeit also genetically distinct from, the IP populations based on *F*_*ST*_, SAMOVA and DAPC analyses of microsatellite data. However, STRUCTURE results were congruent with mitochondrial data. While we cannot fully account for the phylogeographic pattern observed, the genetic differentiation inferred from microsatellite analyses of both species essentially reflects the relative geographic distances between the Maldives, CK and Western Australia, and, more importantly, implies that the CK population of both species was in effect isolated from the majority of CIP and WIP stocks at an ecological time scale.

### Influence of species-specific features

A number of features could have, perhaps in concert, resulted in the drastically different phylogeographic patterns observed in the three grouper species. A longer evolutionary history could have allowed population differentiation to build up and intensify the genetic signal. The divergence of the *P. areolatus* WAPO lineage from the rest was much more recent (late Pleistocene) than divergence of the *P. leopardus* East and West lineages (early Pleistocene), which might partly explain the weak population structure observed in the former species. Nonetheless, length of evolutionary history alone cannot explain the similarly weak population structure observed in *E. polyphekadion*, whose WIP lineage also emerged in the early Pleistocene. The much lower dispersal ability of *P. leopardus* in comparison to the other species is the most probable explanation for the disparity observed. In fact, the smaller distribution range of *P. leopardus* might be an indication that this species is less capable of long distance dispersal.

The patterns of reproductive output per spawning event at each FSA differ substantially among the three species studied, leading to possible differences in connectivity linked to larval dispersal. Spawning aggregations of *E. polyphekadion* and *P. areolatus* in unexploited or lightly exploited areas can involve thousands of reproductive adults at fewer sites, with spawning occurring in reef passages adjacent to the open ocean around the full or new moon periods. In contrast, *P. leopardus* FSAs usually consist of a few hundred adults [38] at multiple sites, spawning down current during medium to strong current flows associated with the new moon (at least in Australia) [24]. Hence, massive concentrated pulses of gametes are generated for the former two (reef passage-spawning) species, while *P. leopardus* aggregations generate an order of magnitude smaller and much more spatially distributed series of gamete source pulses within an area. Given that the three species have similar PLDs, and that monthly spawning events for all three species are similarly brief, often during times when tidal flows are greatest (full and/or new moon phases), one might expect the immense number of gametes released by *E. polyphekadion* and *P. areolatus* FSAs to have a greater probability of long-distance dispersal due to oceanography compared to the lower number of propagules released in dispersed smaller pulses (smaller groups of adults). Moreover, *E. polyphekadion* and *P. areolatus* aggregate next to the open ocean where currents can potentially carry gametes long distances from the aggregation area, which contrasts with the inshore/reef platform spawning locations of *P. leopardus*. The dispersal of a few concentrated larger cohorts could potentially be more strongly influenced by meso-scale oceanographic events than multiple, scattered, smaller cohorts, which would probably be more affected by short-term localized events.

On the other hand, given that *P. areolatus* spawn over multiple months while *E. polyphekadion* and *P. leopardus* only spawn for one or two months each year, we might predict wider overall dispersal in *P. areolatus* than in the other two species if oceanographic conditions vary across seasons and thereby spread offspring more widely. More/less extensive larval dispersal may be linked to weaker/stronger population genetic structure, respectively as has been documented in many terrestrial and aquatic (including marine) species [84, 85]. However, dispersal ability more broadly, like PLD *per se,* is not a good predictor of population genetic structure [86] or range size [87] in the marine realm. Maybe the sheer number and concentration of gametes released into strong currents, and possibly the timing and location of these releases, contribute to the increased range of dispersal between populations and the distributional range size of both *E. polyphekadion* and *P. areolatus*.

Few other studies have examined population connectivity in groupers and considered the possible effect of reproductive mode with similar outcomes to our study. Portnoy et al. tested the hypothesis that aggregative spawning behaviour affects gene flow by examining population genetic structure and connectivity of two groupers: the aggregate spawning *Epinephelus guttatus* and the non-aggregating *Cephalopholis fulva* [88]. Over a relatively small geographical scale in the Caribbean Sea, they found weaker population genetic structure in the aggregate-spawner, contrary to the expectation of the authors that such a reproductive mode would lower the genetic exchange between catchment areas of spawning aggregations. This result was partly attributed by the authors to the fact that *C. fulva* is sedentary, spawning over large areas of the shallow reef platform, and may maintain the same territory, forming multiple small mating groups over multiple months in the year [89]. Additionally, Portnoy et al. proposed that *E. guttatus*, which aggregates over just a few months each year and at the shelf drop off close to open ocean, may join other aggregations in subsequent (unexamined) years, thereby homogenizing the gene pool, given that *E. guttatus* can migrate more than 30 km to spawning sites. This proposal, however, does not consider pelagic larval distribution and recruitment as an alternative avenue of connectivity. Consistent with this finding, *E. striatus*, which forms large spatially and temporally restricted spawning aggregations like *E. guttatus* in the Caribbean, was also found to exhibit weak population genetic structure across a long distance (~ 1600 km) [90]. Results from parentage analysis of this species suggested significant external recruitment caused by high population connectivity [90].

In summary, stronger genetic structuring appears to be closely associated with species that form multiple small spawning groups away from the shelf edge (e.g. *P. leopardus* and *C. fulva*). Reduced population structuring is evident among species that assemble in large numbers at relatively few spawning sites at the shelf edge close to the open ocean (e.g. *E. polyphekadion, E. guttatus, E. striatus* and *P. areolatus*) (Additional file 1: Figure S7). We have assumed that the population sizes (both historical and present) of the three species are comparable, and that the aggregation type for each species remains invariant throughout their distributional ranges. Given their similar habitat and dietary requirements, and considering that all are considered to be common enough to exploit throughout their ranges, the first assumption is reasonable. While the latter assumption has not been comprehensively assessed because data on aggregations on all three species are not available throughout their ranges, all available publications show that this assumption holds true (including where they co-distribute) and where comparisons have been possible (e.g. [91]; database from www.scrfa.org), fully supporting our approach. Nonetheless, further studies on reproductive biology and spawning behaviour of these species are encouraged to advance our understanding of factors shaping population connectivity. Such information could also provide important information for fishery management frameworks for these economically important reef fishes [92]. Although other regionally relevant factors, such as retention of larvae at natal grounds in some locations, are known to occur (e.g. *P. maculatus* [93], and *P. areolatus* [94]), such studies of self- or local recruitment were performed on single cohorts of recruits from a limited part of the distributional range, using parentage analyses. Hence, these findings are unlikely to contradict the connectivity measured here over evolutionary time scales by sampling mixed age adult cohorts from across the range. While our study suggests an association between reproductive mode and population structure, to what extent this factor impacts connectivity at ecological time scales is largely unknown. Nonetheless, our results highlight the need to consider the possible influence of reproductive mode (spatial and temporal aspects of reproduction) in future studies of population genetics and connectivity.

### Conservation and management implications

Groupers are highly sought after and many are also often intrinsically vulnerable to fishing pressure because of their longevity, late sexual maturation, high site fidelity, complex social structure and, for some species, protogynous hermaphroditism [95–97]. As many as 25% of all exploited epinepheline species may be at risk from fishing activities or coastal development [98]. Species that form spawning aggregations heavily exploited by aggregation-fisheries may be particularly at risk because such reproductive gatherings of spawning fish tend to be temporally and spatially predictable and are easily and quickly fished out once discovered and if not suitably managed [28]. There are declines or disappearances of *E. polyphekadion* and *P. areolatus* spawning aggregations in Palau, Fiji, Pohnpei, French Polynesia and Indonesia for example, (www.scrfa.org). Our study species are listed as threatened or near-threatened on the IUCN Red List, largely as a result of uncontrolled aggregation-fishing [99]. Better fishery management and conservation (e.g. by marine protected areas (MPAs), seasonal protection from fishing during spawning, or, ideally, some combination) are urgently needed [98].

The results of this study highlight the importance of population structuring in determining possible management units and the spatial scale that management would need to consider. For example, species with large catchment areas like *E. polyphekadion* and *P. areolatus* would need larger MPAs and regional scale management than a species like *P. leopardus*. Conversely, other management methods may be more practical for the former two species such as seasonal protection during spawning complemented by seasonal sales bans because enforcement may be particularly challenging for offshore sites [92]. However, connectivity analyses at smaller scales have also revealed high self- or local recruitment within a single cohort sometimes justifying the benefit of more local scale management strategies (e.g. *P. maculatus* [93], and *P. areolatus*, [94]). Taken together, both regional- and local-scale management may need to be considered to ensure sustainable fisheries of these economically important reef fishes.

## Conclusions

This study identified phylogeographic signatures of Pleistocene glacial cycles in all three species, with genetic breaks generally coinciding with major biogeographic boundaries, while species-specific reproductive traits determine the magnitude of these phylogeographic signals. As hypothesized, *P. leopardus* exhibited markedly stronger population genetic structure at various geographic scales within the CIP than the other two groupers, while *E. polyphekadion* displayed only slightly stronger population structuring than *P. areolatus*. The populations in the CIP were generally highly connected in the latter two species, and a significant genetic break only occurred among ocean basins. Results from this study and a comparable study in the Caribbean both suggested that stronger genetic structuring appeared to be closely associated with species that form multiple small spawning groups on the shelf platform and away from the open ocean. Our results highlighted the need for more investigations on this characteristic and the need to consider reproductive mode in studies of connectivity and population genetics as well as in more sustainable fisheries management.

## Additional file

Additional file 1: Additional details of sampling, methodology, and results: **Table S1.** Primers used for PCR amplification of control region sequences. **Table S2-S8.** Basic information and genetic diversity of control region and microsatellite datasets. **Table S9.** SAMOVA results. **Table S10-S15.** Pairwise *Φ*_*ST*_ and *F*_*ST*_ of control region and microsatellite datasets. **Table S16.** Results of linkage disequilibrium test. **Figure S1-S6.** Results of DAPC analyses. **Figure S7.** Summary of population genetic structure and reproductive characteristics of five grouper species analysed in this study and Portnoy et al. (2013). (PDF 4110 kb)

## Abbreviations

CIOI: Central Indian Ocean Islands
CIP: Central Indo-Pacific
DAPC: Discriminant analysis of principal components
EIP: Eastern Indo-Pacific
FSA: Fish spawning aggregation
IP: Indo-Polynesian
IPB: Indo-Pacific Barrier
MPA: Marine Protected Area
MSN: Minimum spanning network
mtDNA: Mitochondrial DNA
PLD: Pelagic larval durations
RS: Red Sea
RSB: Red Sea Barrier
WAPO: Western Australia-Pacific Ocean
WIP: Western Indo-Pacific
