# Supplementary material for: Contrasting population genetic structure in three aggregating groupers (Percoidei: Epinephelidae) in the Indo-West Pacific: the importance of reproductive mode
Source: BMC Evol Biol. 2018 Dec 4;18:180. doi: 10.1186/s12862-018-1284-0 (PMC6278153; doi:10.1186/s12862-018-1284-0)
Supplement: Supplementary file 1 — Additional details of sampling, methodology, and results: Table S1. Primers used for PCR amplification of control region sequences. Table S2-S8. Basic information and genetic diversity of control region and microsatellite datasets. Table S9. SAMOVA results. Table S10-S15. Pairwise ΦST and FST of control region and microsatellite datasets. Table S16. Results of linkage disequilibrium test. Figure S1-S6. Results of DAPC analyses. Figure S7. Summary of population genetic structure and reproductive characteristics of five grouper species analysed in this study and Portnoy et al. (2013). (PDF 4110 kb) [file 12862_2018_1284_MOESM1_ESM.pdf]

Additional file 1

Additional details of sampling, methodology, and results

Table S1. Primers used for PCR amplification of control region sequences.

Primer	Direction	Sequence (5'-3')	Species	Source
EP CR F2	Forward	AGCATATATACGGTTCAACCATTC	*E. polyphekadion*	This study
12S R	Reverse	GCGGAGGCTTGCATGTGTA	*E. polyphekadion*	1
ThrF	Forward	TCAAAACGACGGTCTTGTAA	*P. areolatus, P. leopardus*	This study
CR-PA-R	Reverse	TTCTTGCTAGGTGGGTAACGAGT	*P. areolatus, P. leopardus*	This study

[1] Miya M, Nishida M. Organization of the mitochondrial genome of a deep-sea fish, *Gonostoma gracile* (Teleostei: Stomiiformes): first example of transfer RNA gene rearrangements in bony fishes. Mar Biotech. 1999; 1: 416-426.

Table S2. *Epinephelus polyphekadion* control region (723 bp) statistics required for estimating genetic diversity (h and π).

Sampling sites	Code	N	Na	h	h stdev	π	π stdev	τ	τ 2.5%	τ 97.5%
Pacific Ocean + Eastern Indian Ocean*	EIWP	243	156	0.992	0.002	0.024	0.012	6.730	3.221	26.018
French Polynesia	TM	16	4	0.350	0.148	0.001	0.001	0.445	0.000	1.107
Fiji	FJ	35	30	0.992	0.009	0.021	0.011	6.926	3.238	19.416
New Caledonia	NC	29	21	0.966	0.022	0.023	0.012	3.180	0.639	28.342
Pohnpei	PO	17	13	0.963	0.033	0.025	0.013	17.504	4.238	27.527
Palau	PL	38	33	0.992	0.009	0.027	0.014	18.037	10.336	29.004
Philippines	PH	33	27	0.989	0.010	0.025	0.013	21.248	12.393	25.705
+Dumaguete, Philippines^	PD	8^	8	1.000	0.063	0.023	0.013	19.547	9.932	24.482
+Cebu, Philippines	PC	19^	18	0.994	0.019	0.028	0.014	22.027	15.648	25.652
+Palawan, Philippines^	PW	6^	5	0.933	0.122	0.020	0.012	0.000	0.000	22.820
Okinawa[§]	OK	2[§]	2	1.000	0.500	0.027	0.028	0.000	0.000	0.000
Bangkok[§]	TH	2[§]	11	1.000	0.500	0.021	0.022	0.000	0.000	0.000
Western Australia	WA	34	25	0.977	0.014	0.024	0.012	22.715	11.504	28.061
+Scott Reef, Western Australia	SR	16	15	0.992	0.025	0.027	0.014	22.787	13.641	27.072
+Rowley Shoals, Western Australia	CR	18	12	0.941	0.039	0.020	0.010	5.898	1.824	28.105
Cocos (Keeling) Islands	CK	36	20	0.948	0.020	0.028	0.014	30.236	1.186	37.240
Indian Ocean	IO	27	23	0.989	0.013	0.026	0.013	12.273	5.990	32.676
Phuket[§]	PK	4[§]	3	0.833	0.222	0.023	0.016	14.363	5.957	28.105
Maldives	ML	23	21	0.992	0.015	0.027	0.014	11.225	4.998	37.098
**All**		**270**	**179**	**0.993**	**0.002**	**0.050**	**0.024**	**6.715**	**2.918**	**29.471**

N: number of individuals analysed; Na: number of haplotypes; h: haplotype diversity, π: nucleotide diversity; τ 2.5% and 97.5%: 95% CI of Tau.

* Eastern Indian Ocean includes Western Australia and Cocos Keeling in this dataset, as population genetics analyses find these two populations are more closely related to conspecifics in the Pacific Ocean than other Indian Ocean populations.

^Two of the Philippines populations have small sample sizes, all three Philippines populations were pooled in all subsequent analyses.

[§] Three populations have small sample sizes and are excluded from SAMOVA.

Table S3. *Plectropomus areolatus* control region (580 bp) statistics required for estimating genetic diversity (h and π).

Sampling sites	Code	N	Na	h	h stdev	π	π stdev	τ	τ 2.50%	τ 97.50%
Pacific Ocean + Western Australia	PAC	196	44	0.813	0.027	0.004	0.002	2.633	0.592	4.570
Fiji	FJ	21	11	0.848	0.070	0.003	0.002	1.910	0.609	2.986
Solomon Islands	SI	15	5	0.705	0.088	0.003	0.002	2.480	0.000	4.969
Torres Strait	TS	25	11	0.840	0.058	0.004	0.003	2.748	0.939	4.518
Pohnpei	PO	28	9	0.762	0.073	0.003	0.002	2.547	0.195	5.270
Palau	PL	15	7	0.800	0.083	0.004	0.002	2.668	0.607	4.902
Philippines (Cebu)	PC	32	13	0.875	0.042	0.004	0.003	2.873	1.068	4.627
Indonesia	INS	22	11	0.905	0.043	0.005	0.003	2.719	1.379	3.781
+Bali, Indonesia^	BA	6^	5	0.933	0.122	0.006	0.004	1.596	0.000	3.178
+Borneo, Indonesia^	BO	1^	1	1.000	0.000	0.000	0.000	0.000	0.000	0.000
+East Indonesia	IN	15	9	0.924	0.044	0.004	0.003	3.162	1.441	4.490
Western Australia	WA	38	13	0.664	0.087	0.003	0.002	3.182	0.000	6.656
+Scott Reef, Western Australia	SR	18	8	0.641	0.130	0.003	0.002	0.000	0.000	0.473
+Rowley Shoals, Western Australia	CR	20	7	0.690	0.105	0.003	0.002	2.688	0.000	5.527
Indian Ocean	IO	65	17	0.873	0.030	0.004	0.002	2.695	1.105	4.313
Cocos (Keeling) Islands	CK	50	10	0.835	0.035	0.004	0.002	2.500	0.496	4.480
Maldives	ML	15	9	0.924	0.044	0.004	0.003	2.635	1.010	4.057
Red Sea	RS	80	38	0.752	0.055	0.003	0.002	2.217	0.277	4.836
Jizan, Red Sea	JI	26	12	0.717	0.098	0.002	0.001	1.242	0.455	2.227
Thuwal, Red Sea	TU	26	11	0.674	0.104	0.004	0.002	0.000	0.000	0.479
Umluj, Red Sea	UM	28	17	0.852	0.067	0.004	0.002	2.166	0.809	3.566
**All**		**341**	**99**	**0.920**	**0.010**	**0.025**	**0.013**	**344.000**	**344.000**	**344.000**

N: number of individuals analysed; Na: number of haplotypes; h: haplotype diversity, π: nucleotide diversity; τ 2.5% and 97.5%: 95% CI of Tau.
^Two of the Indonesian populations have small sample sizes and are excluded from SAMOVA.

Table S4. *Plectropomus leopardus* control region (556 bp) statistics required for estimating genetic diversity (h and π).

Sampling sites	Code	N	Na	h	H stdev	π	π stdev	τ	τ 2.50%	τ 97.50%
Eastern Population*	East	97	58	0.933	0.021	0.052	0.025	36.176	10.041	43.387
Fiji	FJ	30	7	0.366	0.112	0.001	0.001	1.895	0.000	4.301
New Caledonia	NC	16	10	0.867	0.079	0.020	0.011	9.352	3.846	20.117
Solomon Islands	SI	12	12	1.000	0.034	0.016	0.009	3.281	0.352	9.424
Capricorn Bunker Group, QLD	CB	19	16	0.983	0.022	0.029	0.015	4.871	1.232	28.543
Torres Strait	TS	20	18	0.990	0.019	0.025	0.013	2.428	0.393	15.838
Western Population*	West	243	125	0.980	0.004	0.040	0.020	28.453	8.719	33.613
Indonesia	INS	23	15	0.917	0.044	0.038	0.019	27.914	7.836	33.678
+East Indonesia	EI	17	13	0.927	0.058	0.040	0.021	28.375	6.090	34.307
+Borneo^	BO	3^	3	1.000	0.272	0.033	0.025	4.225	1.055	22.834
+Bali^	BA	3^	2	0.667	0.314	0.010	0.008	7.982	0.813	92.982
Taiwan	TW	24	19	0.971	0.024	0.030	0.016	22.764	4.172	94.432
Palau	PL	29	15	0.872	0.053	0.011	0.006	2.369	0.000	12.447
Philippines	PH	38	20	0.918	0.026	0.026	0.013	21.480	7.385	28.363
+Palawan, Philippines	PW	17	9	0.846	0.070	0.015	0.008	9.908	1.164	15.756
+Cebu, Philippines	PC	21	14	0.895	0.061	0.018	0.010	1.943	0.000	18.604
Western Australia	WA	129	69	0.964	0.010	0.024	0.012	12.533	2.637	16.803
+Scott Reef, Western Australia	SR	87	47	0.939	0.019	0.015	0.008	10.344	1.938	14.924
+Abrolhos Islands, Western Australia	AR	42	23	0.940	0.022	0.031	0.016	14.305	9.254	17.678
**ALL**		**340**	**183**	**0.984**	**0.003**	**0.131**	**0.063**	**26.133**	**8.443**	**30.484**

N: number of individuals analysed; Na: number of haplotypes; h: haplotype diversity, π: nucleotide diversity; τ 2.5% and 97.5%: 95% CI of Tau.

* Eastern and Western Group based on control region minimum spanning network analysis.

^Two of the Indonesian populations have small sample sizes and are excluded from SAMOVA.

Table S5. Basic information of microsatellite loci used in this study.

Loci	F_{ST} not using ENA	F_{ST} using ENA	Null allele^	Forward primer (5'-3')	Reverse primer (5'-3')	Repeats	Reference
E. polyphekadion							
EP11	0.230	0.230		AGGGAACACAGGCGTATGG	TGACCCAGTCAACCTGCAC	GAT	1
EP28	0.321	0.320		GACGTTTCAAATGGCGATGC	ACACACAGTGATTATGCTTGG	GT	1
Ep25	0.141	0.141	FJ, NC, PH, PL, TM, CK	AGCACTTTGTGGTTTCTACCTG	TGTGCCCAAGATGAATTTCCC	ACTT	1
EP33	0.055	0.053	ML, NC, PH, PL, PO	TCTGTGGGAGCTTAAGACCG	AGGAACTGTGTCCAGTAGCG	AC	1
EP09	0.015	0.013		CCGACATCAACTACCTTTCCG	TCTGTGTCACGCCGACC	CTGT	1
EP14	0.097	0.090	NC, PH	TGCAGGTAGAACTCAGGGC	TGCCGCTAGATCACAGCTC	ATC	1
EP35	0.051	0.050	FJ, PH	GCTCAAAGTCACGTGCCAG	AAACCACCATGTTGCGATG	AGAT	1
EP34	0.035	0.035	NC	CTGCCAACCATTTCACAAAGC	GGTGTTGGTGTTTCCTTCCAC	ATCT	1
EP02	0.268	0.269		ACCTCAGGAAGAGCAACTG	CAAAGAGGAGATCGGCATCG	ACAG	1
EP21	0.152	0.152	PH	CCACCATCTCTTTGAACCGTG	GCCGACCTTGGACGTTAATC	CT	1
EP20	0.140	0.151	NC, PL, PO, TM, CK	ACTTGGACTCAGGTTTCCC	GCAGGTCACATGACTACCG	AC	1
**All Loci**	0.121	0.122					
**Bootstrap 95%CI**	0.078-0.174	0.077-0.175					
P. areolatus							
Pm5	0.215	0.208	ML, PH, PL, SR	TCAAGGGACACAGAAATGGTTCA	CCTGCGCTGACCCTGTTTTA	CA	2
Pm3	0.123	0.118	ML, CK	AGCTGCATAAGCACTTACCGTCACTG	CACGACCCCCAGAGCAAACAC	CA	2
PaD2	0.358	0.354		CTCAGCACAATGGGTGGTTA	CTCAGCACAATGGGTGGTTA	TAGA	3
Pb111	0.251	0.247		GTCAGTGTCCCGGTTTGTCT	GAGGTTTCACCGGAAGAACA	TTG	3
Pb120	0.224	0.223		AGAAGAAGTTGGCCATGACG	TGTGTGTAAGACCCACAGGAA	CAA	3
Pa114	0.164	0.164		ATCCTCCTCCCGGTAACAAC	GTTTGTCTGCCGGTGAATTT	TG, GA	3
Pa117	0.204	0.204		ATATCCGCCCTGTTCACATC	GTAATGGCCCTGCTTTGTGT	CA	3
Pm6	0.116	0.112	CK	AGCTACTGTCCGCCTCTGTTAATGCTA	AACAGGATGCTGAAATAGAATTTGG	CA	2
Pma038	0.229	0.223	FJ, TS	TATGGAGGGATGATGCTATCTAAGAG	ATGCTAAACTGGATGCACTACAATC	AGAT	4
**All Loci**	0.204	0.201					
**Bootstrap 95%CI**	0.164-0.249	0.161-0.246					
P. leopardus							
Pma101	0.120	0.120		TGTTCTGTCAGATATGTAATGTGCTG	GGGGATAGACAAGAGGAAAGAGAGGGGA	TATC	4
Pma109	0.234	0.246	LI	TGCCCGACTCGATTTGTAACAGTGC	ACTCAGATATCTTGAGGTTAGAGGTC	ATCT	4
Pma106	0.099	0.087	SR, AR, PL, PH, TW, LI, FJ	CAGGAGCCATTGAGACAGGGAGAGG	AGTGTTGGTGGTTTCGCTGATGCTT	GATA	4
Ple002	0.088	0.079	PH, LI	TACTCGCAATTATAACACAGATCCAG	TTTGTCCAGCACTGTATTTATCTATC	AGAT	4
Pma090	0.209	0.202	FJ	GATGTCCAAATATCACCTCTAACCAG	AGAGGCTCAATATTATCATGTGAACG	TAGA	4
Pma112	0.105	0.091	SR, AR, PL, PH, LI, NC, FJ	CTGCACTTTAATACCCATGAAATAGC	TGGAAACCAGTTAAATAATCCCTGAC	TATC	4
Pm3	0.101	0.094	SR, AR, PH, TW, FJ	AGCTGCATAAGCACTTACCGTCACTG	CACGACCCCCAGAGCAAACAC	CA	2
Pma022	0.229	0.231	SR, AR, TW	AAGATGTGCACTGTCAATACACTATG	GATGTCAGATATCAGGCTCCTAAATG	AGAT	4
Ple005	0.106	0.101	SR, AR, PL, PH, TW	AACTACAATGAAACCTGCCTCTTATG	TTTGATTATGACTCAATGATCGCAAG	ACAG	4
**All Loci**	0.140	0.135					
**Bootstrap 95%CI**	0.109-0.180	0.100-0.179					

^ Populations with null alleles detected by MICROCHECKER

1 Ma KY, de Mitcheson YS, Chu KH. Isolation and characterization of microsatellite markers from the camouflage grouper, *Epinephelus polyphekadion* (Epinephelidae). Conserv Genet Resour. 2013; 5:1129-1132.

2 Zhu ZY, Lo LC, Lin G, Xu YX, Yue GH. Isolation and characterization of polymorphic microsatellites from red coral grouper (*Plectropomus maculatus*). Mol Ecol Resour. 2005;5:579-581.

3 Almany GR, Hamilton RJ, Bode M, Matawai M, Potuku T, Saenz-Agudelo P, Plane, S, Berumen ML, Rhodes KL, Thorrold SR, Russ GR. Dispersal of grouper larvae drives local resource sharing in a coral reef fishery. Curr Biol. 2013;23:626-630.

4 Harrison HB, Feldheim KA, Jones GP, Ma K, Mansour H, Perumal S, Williamson DH, Berumen, ML. Validation of microsatellite multiplexes for parentage analysis and species discrimination in two hybridizing species of coral reef fish (*Plectropomus* spp., Serranidae). Ecol Evol. 2014;4:2046-2057.

Table S6. *Epinephelus polyphekadion* basic genetic diversity statistics based on 11 microsatellite loci.

**Loci**			**EP11**				**EP25**				**EP09**				**EP35**				**EP02**				**EP20**			
**Sampling sites**	**Code**	**N**	**Na**	**Ho**	**He**	**adjP**	**Na**	**Ho**	**He**	**adjP**	**Na**	**Ho**	**He**	**adjP**	**Na**	**Ho**	**He**	**adjP**	**Na**	**Ho**	**He**	**adjP**	**Na**	**Ho**	**He**	**adjP**
Pacific Ocean + Eastern IO	PAC	221	-	-	-	-	-	-	-	-	-	-	-	-	-	-	-	-	-	-	-	-	-	-	-	-
French Polynesia	TM	17	2	0.118	0.111	1.000	5	0.294	0.728	0.000	3	0.647	0.455	1.000	11	0.647	0.730	0.285	2	0.059	0.057	1.000	5	0.471	0.740	0.003
Fiji	FJ	33	1	0.000	0.000	1.000	7	0.394	0.778	0.000	4	0.515	0.424	1.000	15	0.577	0.887	0.000	5	0.333	0.296	1.000	6	0.606	0.680	0.136
New Caledonia	NC	30	2	0.200	0.180	1.000	8	0.667	0.799	0.009	4	0.467	0.410	1.000	11	0.731	0.836	0.028	5	0.433	0.542	0.360	6	0.552	0.764	0.013
Pohnpei	PO	25	2	0.040	0.039	1.000	6	0.560	0.723	0.047	4	0.240	0.220	1.000	16	0.875	0.909	0.094	5	0.708	0.584	1.000	6	0.560	0.755	0.015
Palau	PL	39	3	0.316	0.278	1.000	8	0.333	0.762	0.000	4	0.231	0.255	0.100	14	0.838	0.876	0.285	3	0.667	0.534	1.000	7	0.538	0.789	0.000
Philippines	PH	22	1	0.000	0.000	1.000	5	0.476	0.686	0.031	3	0.182	0.244	0.100	13	0.550	0.894	0.000	2	0.500	0.455	1.000	5	0.650	0.685	0.236
+Cebu, Philippines	PC	17	-	-	-	-	-	-	-	-	-	-	-	-	-	-	-	-	-	-	-	-	-	-	-	-
+Palawan, Philippines^	PW	5^	-	-	-	-	-	-	-	-	-	-	-	-	-	-	-	-	-	-	-	-	-	-	-	-
Scott Reef, Western Australia	WA	16	3	0.125	0.119	1.000	6	0.563	0.752	0.031	4	0.375	0.326	1.000	3	0.000	0.625	0.054	4	0.467	0.589	0.893	7	0.667	0.762	0.034
Cocos (Keeling) Islands	CK	39	3	0.256	0.231	1.000	8	0.583	0.728	0.031	4	0.250	0.271	0.713	11	0.813	0.803	0.179	5	0.737	0.558	1.000	8	0.605	0.810	0.006
Indian Ocean	IO	40	-	-	-	-	-	-	-	-	-	-	-	-	-	-	-	-	-	-	-	-	-	-	-	-
Maldives	ML	40	5	0.692	0.537	1.000	6	0.575	0.478	0.995	5	0.375	0.399	0.509	14	0.828	0.885	0.028	2	0.175	0.160	1.000	3	0.075	0.073	1.000

Loci			EP28				EP33				EP14				EP34				EP21			
Sampling sites	Code	N	Na	Ho	He	adjP	Na	Ho	He	adjP	Na	Ho	He	adjP	Na	Ho	He	adjP	Na	Ho	He	adjP
Pacific Ocean + Eastern IO	PAC	221	-	-	-	-	-	-	-	-	-	-	-	-	-	-	-	-	-	-	-	-
French Polynesia	TM	17	2	0.059	0.057	1.000	6	0.824	0.766	0.419	4	0.588	0.678	0.214	9	0.765	0.742	0.899	4	0.529	0.528	0.722
Fiji	FJ	33	3	0.212	0.195	1.000	15	0.758	0.810	0.146	6	0.742	0.696	0.791	11	0.806	0.868	0.000	8	0.467	0.552	0.499
New Caledonia	NC	30	4	0.207	0.191	1.000	12	0.577	0.848	0.005	7	0.538	0.727	0.114	12	0.655	0.846	0.071	4	0.533	0.529	0.347
Pohnpei	PO	25	4	0.280	0.314	1.000	11	0.583	0.864	0.001	6	0.667	0.699	0.499	10	0.760	0.827	0.181	4	0.684	0.633	0.843
Palau	PL	39	3	0.211	0.194	1.000	11	0.429	0.777	0.000	7	0.667	0.725	0.114	11	0.829	0.888	0.181	5	0.564	0.503	0.848
Philippines	PH	22	2	0.091	0.087	1.000	12	0.474	0.868	0.000	5	0.313	0.664	0.032	12	0.895	0.863	0.899	4	0.278	0.551	0.031
+Cebu, Philippines	PC	17	-	-	-	-	-	-	-	-	-	-	-	-	-	-	-	-	-	-	-	-
+Palawan, Philippines^	PW	5^	-	-	-	-	-	-	-	-	-	-	-	-	-	-	-	-	-	-	-	-
Scott Reef, Western Australia	WA	16	1	0.000	0.000	1.000	10	0.714	0.855	0.054	7	0.833	0.774	0.841	9	0.938	0.871	0.899	4	0.462	0.476	0.722
Cocos (Keeling) Islands	CK	39	2	0.108	0.102	1.000	9	0.741	0.776	0.299	8	0.769	0.778	0.160	12	0.895	0.877	0.468	6	0.538	0.700	0.329
Indian Ocean	IO	40	-	-	-	-	-	-	-	-	-	-	-	-	-	-	-	-	-	-	-	-
Maldives	ML	40	10	0.641	0.696	1.000	9	0.333	0.535	0.004	7	0.825	0.678	0.992	10	0.914	0.841	0.899	2	0.475	0.447	0.843

N: number of individuals analysed; Na: mean number of alleles; He: expected heterozygosity; Ho: observed heterozygosity; yellow highlighted adjP: p-value of HWE test adjusted by Benjamini–Hochberg procedure < 0.01.

^Two Philippines populations have small sample sizes and both populations were pooled for all subsequent analyses.

Table S7. *Plectropomus areolatus* basic genetic diversity statistics based on 9 microsatellite loci.

Loci			Pm5				PaD2				Pb120				Pa117				Pma038			
**Sampling sites**	**Code**	**N**	**Na**	**Ho**	**He**	**adjP**	**Na**	**Ho**	**He**	**adjP**	**Na**	**Ho**	**He**	**adjP**	**Na**	**Ho**	**He**	**adjP**	**Na**	**Ho**	**He**	**adjP**
Pacific Ocean + Western Australia	PAC	227	-	-	-	-	-	-	-	-	-	-	-	-	-	-	-	-	-	-	-	-
Fiji	FJ	28	8	0.609	0.749	0.036	3	0.250	0.223	1.000	6	0.571	0.527	1.000	5	0.500	0.543	1.000	3	0.321	0.469	0.154
Solomon Islands	SI	30	6	0.867	0.688	0.997	4	0.367	0.396	0.936	5	0.500	0.407	1.000	4	0.567	0.536	1.000	3	0.567	0.535	0.821
Torres Strait	TS	27	8	0.741	0.804	0.219	3	0.296	0.426	0.670	5	0.500	0.498	1.000	2	0.556	0.431	1.000	5	0.423	0.697	0.001
Pohnpei	PO	42	9	0.667	0.720	0.694	5	0.238	0.253	0.936	5	0.714	0.614	1.000	6	0.667	0.576	1.000	8	0.619	0.615	0.154
Palau	PL	19	7	0.474	0.733	0.006	3	0.316	0.373	0.936	4	0.579	0.528	1.000	5	0.526	0.496	1.000	7	0.313	0.467	0.160
Philippines (Cebu)	PC	35	6	0.571	0.711	0.157	3	0.294	0.397	0.670	5	0.600	0.613	0.961	4	0.486	0.436	1.000	6	0.636	0.578	0.489
East Indonesia^	EI	10^	6	0.700	0.780	0.504	3	0.500	0.485	1.000	3	0.800	0.535	1.000	3	0.800	0.535	1.000	3	0.600	0.565	0.821
Western Australia	WA	36	-	-	-	-	-	-	-	-	-	-	-	-	-	-	-	-	-	-	-	-
+Scott Reef, Western Australia	SR	13	5	0.308	0.583	0.006	3	0.385	0.476	0.936	7	0.615	0.607	1.000	6	0.692	0.589	1.000	4	0.385	0.331	1.000
+Rowley Shoals, Western Australia	CR	23	6	0.609	0.542	0.997	4	0.739	0.518	1.000	6	0.826	0.573	1.000	6	0.636	0.490	1.000	6	0.571	0.574	0.821
Indian Ocean	IO	67	-	-	-	-	-	-	-	-	-	-	-	-	-	-	-	-	-	-	-	-
Cocos (Keeling) Islands	CK	55	10	0.691	0.640	0.883	3	0.519	0.401	1.000	5	0.778	0.655	1.000	9	0.909	0.759	1.000	7	0.545	0.562	0.592
Maldives	ML	12	2	0.000	0.444	0.006	1	0.000	0.000	1.000	1	0.000	0.000	1.000	3	1.000	0.538	1.000	5	0.917	0.747	0.993
Red Sea	RS	103	-	-	-	-	-	-	-	-	-	-	-	-	-	-	-	-	-	-	-	-
Jizan, Red Sea	JI	27	4	0.556	0.615	0.587	4	0.615	0.501	1.000	7	0.704	0.737	1.000	14	0.654	0.749	0.445	11	0.917	0.834	1.000
Thuwal, Red Sea	TU	37	3	0.649	0.493	0.997	3	0.622	0.520	1.000	6	0.703	0.702	0.339	14	0.848	0.832	1.000	11	0.811	0.866	0.273
Umluj, Red Sea	UM	39	4	0.462	0.504	0.504	3	0.590	0.489	1.000	6	0.795	0.726	1.000	14	0.763	0.783	0.056	11	0.897	0.844	0.821

			Pm3				Pb111				Pa114				Pm6			
**Sampling sites**	**Code**	**N**	**Na**	**Ho**	**He**	**adjP**	**Na**	**Ho**	**He**	**adjP**	**Na**	**Ho**	**He**	**adjP**	**Na**	**Ho**	**He**	**adjP**
Pacific Ocean + Western Australia	PAC	227	-	-	-	-	-	-	-	-	-	-	-	-	-	-	-	-
Fiji	FJ	28	8	0.607	0.652	0.586	8	0.714	0.663	0.963	6	0.607	0.619	0.885	11	0.857	0.867	0.537
Solomon Islands	SI	30	8	0.600	0.648	0.475	7	0.700	0.656	0.827	7	0.767	0.729	0.885	13	1.000	0.878	1.000
Torres Strait	TS	27	7	0.846	0.708	0.913	5	0.667	0.594	0.963	6	0.852	0.749	0.885	14	1.000	0.813	1.000
Pohnpei	PO	42	8	0.833	0.715	0.981	11	0.690	0.694	0.117	10	0.857	0.784	0.885	17	0.976	0.849	1.000
Palau	PL	19	9	0.684	0.752	0.170	11	0.684	0.751	0.596	5	0.632	0.661	0.885	14	0.895	0.898	0.682
Philippines (Cebu)	PC	35	7	0.600	0.621	0.662	8	0.743	0.676	0.963	10	0.829	0.789	0.885	13	1.000	0.827	1.000
East Indonesia^	EI	10^	7	0.700	0.760	0.586	3	0.600	0.545	0.963	5	0.700	0.640	0.885	8	0.800	0.805	0.883
Western Australia	WA	36	-	-	-	-	-	-	-	-	-	-	-	-	-	-	-	-
+Scott Reef, Western Australia	SR	13	8	0.846	0.778	0.576	6	0.417	0.590	0.271	5	0.846	0.757	0.885	9	0.769	0.787	0.682
+Rowley Shoals, Western Australia	CR	23	9	0.727	0.697	0.283	10	0.826	0.793	0.827	4	0.783	0.680	0.885	10	0.952	0.771	1.000
Indian Ocean	IO	67	-	-	-	-	-	-	-	-	-	-	-	-	-	-	-	-
Cocos (Keeling) Islands	CK	55	7	0.500	0.665	0.000	10	0.574	0.532	0.341	6	0.722	0.688	0.885	11	0.712	0.827	0.000
Maldives	ML	12	8	0.417	0.733	0.008	1	0.000	0.000	1.000	6	0.917	0.778	0.885	8	0.917	0.816	1.000
Red Sea	RS	103	-	-	-	-	-	-	-	-	-	-	-	-	-	-	-	-
Jizan, Red Sea	JI	27	9	0.704	0.762	0.283	4	0.577	0.672	0.243	8	0.667	0.636	0.885	6	0.519	0.536	0.320
Thuwal, Red Sea	TU	37	9	0.757	0.763	0.283	5	0.556	0.581	0.006	7	0.657	0.612	0.885	14	0.703	0.762	0.450
Umluj, Red Sea	UM	39	9	0.795	0.783	0.576	4	0.769	0.568	1.000	5	0.579	0.574	0.885	8	0.676	0.744	0.230

N: number of individuals analysed; Na: mean number of alleles; He: expected heterozygosity; Ho: observed heterozygosity; yellow highlighted adjP: p-value of HWE test adjusted by Benjamini–Hochberg procedure < 0.01.

^The East Indonesia population has a small sample size and was excluded from DAPC, STRUCTURE and SAMOVA analyses.

Table S8. *Plectropomus leopardus* basic genetic diversity statistics based on 9 microsatellite loci.

Loci			Pma101				Pma106				Pma090				Pm3				Ple005			
**Sampling sites**	**Code**	**N**	**Na**	**Ho**	**He**	**adjP**	**Na**	**Ho**	**He**	**adjP**	**Na**	**Ho**	**He**	**adjP**	**Na**	**Ho**	**He**	**adjP**	**Na**	**Ho**	**He**	**adjP**
**East**	EAS	195	-	-	-	-	-	-	-	-	-	-	-	-	-	-	-	-	-	-	-	-
Fiji	FJ	20	6	0.600	0.545	1.000	8	0.313	0.756	0.001	6	0.588	0.782	0.023	8	0.316	0.740	0.000	8	0.583	0.778	0.155
New Caledonia	NC	37	4	0.667	0.579	1.000	9	0.714	0.787	0.097	2	0.429	0.396	0.868	7	0.826	0.819	0.427	11	0.778	0.800	0.524
Great Barrier Reef	GBR	138	-	-	-	-	-	-	-	-	-	-	-	-	-	-	-	-	-	-	-	-
+Hick's Reef	HR	18	5	0.611	0.543	1.000	12	0.765	0.830	0.124	3	0.667	0.563	0.302	7	0.917	0.795	0.730	7	0.733	0.760	0.220
+Lizard Island	LI	47	6	0.778	0.743	1.000	17	0.727	0.897	0.004	4	0.511	0.472	0.868	7	0.860	0.811	0.730	10	0.787	0.793	0.524
+Townsville	TV	35	7	0.742	0.660	1.000	13	0.407	0.873	0.000	4	0.303	0.391	0.036	7	0.774	0.780	0.427	9	0.706	0.805	0.013
+Swains Reef	SW	38	6	0.621	0.747	0.243	17	0.611	0.913	0.000	3	0.471	0.381	1.000	8	0.861	0.716	0.914	8	0.929	0.804	0.931
**West**	WES	170	-	-	-	-	-	-	-	-	-	-	-	-	-	-	-	-	-	-	-	-
Palau	PL	31	8	0.484	0.601	0.097	12	0.500	0.791	0.000	4	0.467	0.592	0.302	8	0.588	0.721	0.138	8	0.333	0.791	0.000
Taiwan	TW	39	6	0.590	0.627	0.243	8	0.500	0.740	0.004	5	0.500	0.587	0.413	10	0.629	0.790	0.000	10	0.405	0.802	0.000
Philippines	PH	36	-	-	-	-	-	-	-	-	-	-	-	-	-	-	-	-	-	-	-	-
+Palawan, Philippines	PW	19^	11	0.737	0.792	0.580	11	0.750	0.871	0.017	6	0.765	0.661	0.868	5	0.400	0.740	0.045	9	0.267	0.749	0.000
+Cebu, Philippines	PC	17^	10	0.636	0.719	0.535	9	0.375	0.859	0.000	6	0.647	0.728	0.464	4	0.250	0.719	0.055	9	0.636	0.781	0.013
Western Australia	WA	64	-	-	-	-	-	-	-	-	-	-	-	-	-	-	-	-	-	-	-	-
+Scott Reef	SR	33	3	0.061	0.059	1.000	8	0.850	0.686	0.965	4	0.469	0.499	0.302	9	0.364	0.802	0.000	6	0.654	0.746	0.319
+Abrolhos Islands	AR	31	7	0.387	0.342	1.000	15	0.600	0.861	0.000	6	0.645	0.730	0.093	10	0.636	0.693	0.053	8	0.607	0.736	0.011

Loci			Pma109				Ple002				Pma112				Pma022			
**Sampling sites**	**Code**	**N**	**Na**	**Ho**	**He**	**adjP**	**Na**	**Ho**	**He**	**adjP**	**Na**	**Ho**	**He**	**adjP**	**Na**	**Ho**	**He**	**adjP**
**East**	EAS	195	-	-	-	-	-	-	-	-	-	-	-	-	-	-	-	-
Fiji	FJ	20	12	0.818	0.901	0.164	9	0.700	0.790	0.366	7	0.462	0.728	0.013	9	0.667	0.836	0.086
New Caledonia	NC	37	6	0.692	0.683	0.642	8	0.857	0.829	0.708	7	0.351	0.685	0.000	2	0.054	0.053	1.000
Great Barrier Reef	GBR	138	-	-	-	-	-	-	-	-	-	-	-	-	-	-	-	-
+Hick's Reef	HR	18	11	0.438	0.881	0.000	8	0.889	0.796	0.834	10	0.375	0.832	0.000	6	0.389	0.380	0.679
+Lizard Island	LI	47	15	0.500	0.895	0.000	14	0.814	0.847	0.549	13	0.568	0.827	0.000	10	0.587	0.594	0.270
+Townsville	TV	35	12	0.294	0.893	0.000	14	0.879	0.877	0.680	9	0.500	0.786	0.001	9	0.559	0.609	0.610
+Swains Reef	SW	38	9	0.417	0.858	0.000	9	0.286	0.469	0.000	12	0.486	0.788	0.000	4	0.162	0.177	0.371
**West**	WES	170	-	-	-	-	-	-	-	-	-	-	-	-	-	-	-	-
Palau	PL	31	3	0.211	0.215	0.625	9	0.714	0.733	0.366	7	0.641	0.788	0.028	8	0.526	0.602	0.170
Taiwan	TW	39	8	0.300	0.352	0.167	12	0.774	0.836	0.549	9	0.433	0.795	0.000	12	0.839	0.862	0.122
Philippines	PH	36	-	-	-	-	-	-	-	-	-	-	-	-	-	-	-	-
+Palawan, Philippines	PW	19^	5	0.474	0.428	0.966	11	0.722	0.841	0.195	9	0.632	0.837	0.008	11	0.842	0.859	0.153
+Cebu, Philippines	PC	17^	4	0.400	0.580	0.191	10	0.750	0.846	0.113	8	0.750	0.779	0.080	8	0.647	0.830	0.153
Western Australia	WA	64	-	-	-	-	-	-	-	-	-	-	-	-	-	-	-	-
+Scott Reef	SR	33	4	0.667	0.512	0.973	11	0.857	0.811	0.549	5	0.387	0.544	0.000	8	0.667	0.764	0.170
+Abrolhos Islands	AR	31	6	0.839	0.655	0.973	9	0.759	0.821	0.366	9	0.552	0.818	0.000	9	0.774	0.827	0.086

N: number of individuals analysed; Na: mean number of alleles; He: expected heterozygosity; Ho: observed heterozygosity; yellow highlighted adjP: p-value of HWE test adjusted by Benjamini–Hochberg procedure < 0.01.

^Two Philippines populations have relatively small sample sizes; they were pooled in STRUCTURE analyses but not the others.

Table S9. Population structure results for the three species examined, based on SAMOVA analyses of control region sequences and microsatellite allelic frequencies respectively. The number of groups (K) identified and corresponding grouped locations within eco-regions, with the highest F_{CT} for each species based on each dataset shown. IP: Indo-Pacific, CIP: Central Indo-Pacific, EIP: Eastern Indo-Pacific; definition follows Spalding et al. (2007), and WAPO: Western Australia + Pacific Ocean.

				**Standard AMOVA**		**Locus by locus AMOVA**	
**Datasets**	**Region**	**K**	**Grouping**	**F_{CT}**	**p-value**	**F_{CT}**	**p-value**
Control region							
E. polyphekadion	IP	2	{ML}{CK,CR,SR,PL,PH,PO,NC,FJ,TM}	0.852	0.105	0.852	<0.0001
E. polyphekadion	CIP+EIP	2	{CK,CR,SR,PH,PL,PO,NC,FJ}{TM}	0.219	0.123	0.218	<0.0001
E. polyphekadion	CIP	6	{CK}{CR,NC}{SR,PH}{PL}{PO}{FJ}	0.046	<0.01	0.046	<0.0001
P. areolatus	IP	3	{UM,TU,JI}{ML,CK}{CR,SR,EI,PL,PC,PO,TS,SI,FJ}	0.898	<0.0001	0.898	<0.0001
P. areolatus	WAPO	8	{EI}{SI}{CR}{SR}{TS}{PL,PH}{PO}{FJ}	0.045	<0.05	0.045	<0.05
P. leopardus	CIP	9	{AR}{SR,PL}{EI}{PC,TW}{PW}{TS,CB}{SI}{NC}{FJ}	0.879	<0.0001	0.795	<0.0001
Microsatellite							
E. polyphekadion	IP	2	{ML}{CK,WA,PL,PH,PO,NC,FJ,TM}	0.265	0.129	0.197	<0.0001
E. polyphekadion	CIP+EIP	2	{CK,WA,PL,PH,PO,NC,FJ}{TM}	0.066	0.129	0.038	<0.05
E. polyphekadion	CIP	4	{CK}{WA,PL,PH,PO}{NC}{FJ}	0.051	<0.05	0.033	<0.01
P. areolatus	IP	3	{UM,TU,JI}{ML}{CK,CR,SR,PL,PC,PO,TS,SI,FJ}	0.292	<0.0001	0.290	<0.0001
P. areolatus	WAPO	2	{CR,SR}{PL,PC,PO,TS,SI,FJ}	0.043	<0.05	0.043	<0.05
P. leopardus	CIP	10	{AR}{SR}{PW}{PC}{PL}{TW}{LI,HR,TV}{SW}{NC}{FJ}	0.250	< 0.0001	0.133	< 0.0001

Spalding MD, Fox HE, Allen GR, Davidson N, Ferdaña ZA, Finlayson M, et al. Marine Ecoregions of the World: A Bioregionalization of Coastal and Shelf Areas. Bioscience. 2007;57:573–83.

Table S10. *Epinephelus polyphekadion* pairwise Φ_{ST} values, based on 723 bp control region sequence. Benjamini–Hochberg adjusted $P < 0.01$, italics; Benjamini–Hochberg adjusted P < 0.001, bold and italics.

	French Polynesia	Fiji	New Caledonia	Pohnpei	Palau	Philippines	Okinawa*	Bangkok*	Scott Reef	Rowley Shoals	Cocos (Keeling) Is	Phuket*
Fiji	***0.317***											
New Caledonia	***0.315***	0.025										
Pohnpei	***0.464***	***0.132***	***0.066***									
Palau	***0.290***	***0.064***	0.007	0.019								
Philippines	***0.322***	0.062	0.009	0.022	0.003							
Okinawa*	0.844*	0.045*	0.002*	0.075*	-0.047*	-0.021*						
Bangkok*	0.808*	0.027*	-0.061*	-0.092*	-0.108*	-0.084*	-0.100*					
Scott Reef	***0.385***	0.053	0.004	0.014	-0.004	-0.027	-0.076*	-0.104*				
Rowley Shoals	***0.318***	0.033	0.003	***0.116***	0.029	0.027	0.024*	-0.053*	0.008			
Cocos (Keeling) Is	***0.310***	0.048	0.022	0.044	0.016	0.034	-0.012*	-0.062*	0.023	0.058		
Phuket*	***0.975***	***0.880***	***0.872***	***0.863***	***0.849***	***0.860***	0.861*	*0.873**	***0.850***	***0.879***	***0.845***	
Maldives	***0.909***	***0.866***	***0.861***	***0.852***	***0.844***	***0.851***	***0.846***	***0.851***	***0.845***	***0.862***	***0.842***	0.004*

* Population with small sample size

Table S11. *Epinephelus polyphekadion* pairwise F_{ST} values, based on 11 microsatellite loci. Benjamini–Hochberg adjusted $P < 0.01$, italics; Benjamini–Hochberg adjusted P < 0.001, bold and italics.

	French Polynesia	Fiji	New Caledonia	Pohnpei	Philippines	Palau	Western Australia	Cocos (Keeling) Is
Fiji	***0.047***							
New Caledonia	***0.050***	***0.025***						
Pohnpei	***0.099***	***0.047***	0.012					
Philippines	***0.147***	***0.092***	***0.045***	*0.025*				
Palau	***0.104***	***0.056***	***0.021***	*0.018*	0.018			
Western Australia	*0.038*	0.007	-0.018	-0.034	-0.020	-0.035		
Cocos (Keeling) Is	***0.105***	***0.084***	***0.049***	***0.051***	***0.074***	***0.058***	***0.025***	
Maldives	***0.341***	***0.307***	***0.253***	***0.245***	***0.262***	***0.227***	***0.253***	***0.246***

Table S12. *Plectropomus areolatus* pairwise Φ_{ST} values, based on 580 bp control region sequences. Benjamini–Hochberg adjusted $P < 0.01$, italics; Benjamini–Hochberg adjusted P < 0.001, bold and italics.

	Fiji	Solomon Islands	Torres Strait	Pohnpei	Palau	Philippines	Borneo*	East Indonesia	Bali	Scott Reef	Rowley Shoals	Cocos (Keeling) Is	Maldives	Jizan, Red Sea	Thuwal, Red Sea
Solomon Islands	*0.118*														
Torres Strait	*0.072*	0.030													
Pohnpei	0.025	0.026	0.049												
Palau	0.022	0.138	*0.086*	***0.102***											
Philippines	0.024	0.045	0.033	0.048	-0.010										
Borneo*	-0.895	-0.619	-0.731	-0.868	-0.684	-0.756									
East Indonesia	0.019	0.014	0.007	-0.019	0.065	0.015	-0.837								
Bali	0.023	0.025	-0.043	0.037	-0.023	-0.046	-0.920	-0.011							
Scott Reef	0.037	0.010	0.011	-0.002	0.094	0.035	-0.922	-0.009	0.022						
Rowley Shoals	***0.106***	0.081	0.024	*0.064*	*0.146*	0.064	-0.663	0.057	0.061	0.043					
Cocos (Keeling) Is	***0.794***	***0.801***	***0.766***	***0.797***	***0.777***	***0.760***	0.768*	***0.779***	***0.763***	***0.799***	***0.789***				
Maldives	***0.810***	***0.813***	***0.768***	***0.810***	***0.782***	***0.763***	0.758*	***0.774***	***0.750***	***0.815***	***0.806***	***0.161***			
Jizan, Red Sea	***0.958***	***0.959***	***0.943***	***0.954***	***0.954***	***0.940***	0.965*	***0.949***	***0.953***	***0.959***	***0.957***	***0.944***	***0.950***		
Thuwal, Red Sea	***0.940***	***0.939***	***0.927***	***0.939***	***0.933***	***0.926***	0.933*	***0.929***	***0.926***	***0.940***	***0.939***	***0.933***	***0.930***	-0.004	
Umluj, Red Sea	***0.943***	***0.942***	***0.930***	***0.941***	***0.937***	***0.928***	0.938*	***0.933***	***0.930***	***0.943***	***0.942***	***0.935***	***0.933***	0.004	0.001

* Population with small sample size

Table S13. *Plectropomus areolatus* pairwise F_{ST} values, based on nine microsatellite loci. Benjamini–Hochberg adjusted *P* < 0.01, italics; Benjamini–Hochberg adjusted P < 0.001, bold and italics.

	Fiji	Solomon Islands	Torres Strait	Pohnpei	Philippines	Palau	Indonesia*	Scott Reef	Rowley Shoals	Cocos (Keeling) Is	Maldives	Jizan, Red Sea	Thuwal, Red Sea
Solomon Islands	0.010												
Torres Strait	***0.055***	***0.040***											
Pohnpei	*0.014*	*0.016*	***0.046***										
Philippines	***0.026***	***0.023***	***0.039***	***0.020***									
Palau	0.003	0.013	0.023	0.013	0.008								
Indonesia*	0.022	0.021	***0.060***	0.011	***0.047***	*0.036*							
Scott Reef	***0.049***	***0.047***	***0.065***	***0.060***	***0.048***	***0.047***	0.029*						
Rowley Shoals	***0.075***	***0.066***	***0.061***	***0.071***	***0.059***	***0.053***	***0.036***	0.002					
Cocos (Keeling) Is	***0.102***	***0.099***	***0.121***	***0.085***	***0.101***	***0.096***	***0.096***	***0.142***	***0.153***				
Maldives	***0.422***	***0.387***	***0.366***	***0.365***	***0.369***	***0.378***	***0.386***	***0.391***	***0.374***	***0.339***			
Jizan, Red Sea	***0.334***	***0.321***	***0.306***	***0.306***	***0.311***	***0.304***	***0.300***	***0.307***	***0.303***	***0.310***	***0.372***		
Thuwal, Red Sea	***0.333***	***0.320***	***0.300***	***0.307***	***0.311***	***0.302***	***0.295***	***0.302***	***0.296***	***0.308***	***0.353***	0.009	
Umluj, Red Sea	***0.340***	***0.329***	***0.309***	***0.315***	***0.319***	***0.311***	***0.305***	***0.312***	***0.305***	***0.315***	***0.363***	0.006	-0.005

* Population with small sample size

Table S14. *Plectropomus leopardus* pairwise Φ_{ST} values, based on 556 bp control region sequence. Benjamini–Hochberg adjusted *P* < 0.01, italics; Benjamini–Hochberg adjusted P < 0.001, bold and italics.

	Fiji	New Caledonia	Solomon Islands	Cap. & Bunker	Torres Strait	East Indonesia	Borneo*	Bali*	Palau	Taiwan	Cebu	Palawan	Scott Reef
New Caledonia	***0.894***												
Solomon Islands	***0.938***	***0.715***											
Cap. Bunker	***0.843***	*0.172*	***0.647***										
Torres Strait	***0.869***	***0.319***	***0.660***	0.018									
East Indonesia	***0.956***	***0.903***	***0.906***	***0.889***	***0.899***								
Borneo*	***0.990***	*0.922*	*0.937*	***0.893***	*0.909*	0.065							
Bali*	***0.994***	*0.929*	*0.947*	***0.898***	*0.915*	0.099	-0.018						
Palau	***0.983***	***0.953***	***0.961***	***0.939***	***0.946***	***0.509***	***0.805***	***0.835***					
Taiwan	***0.962***	***0.920***	***0.924***	***0.907***	***0.915***	***0.246***	-0.022	***0.294***	***0.729***				
Cebu	***0.979***	***0.941***	***0.949***	***0.924***	***0.933***	***0.369***	0.233	*0.556*	***0.811***	0.015			
Palawan	***0.981***	***0.939***	***0.948***	***0.920***	***0.930***	*0.221*	0.160	-0.114	***0.812***	***0.360***	***0.535***		
Scott Reef	***0.963***	***0.947***	***0.949***	***0.941***	***0.944***	***0.480***	***0.723***	***0.746***	***0.107***	***0.705***	***0.754***	***0.748***	
Abrolhos Islands	***0.943***	***0.909***	***0.910***	***0.901***	***0.907***	***0.248***	***0.460***	***0.515***	***0.313***	***0.497***	***0.568***	***0.564***	***0.281***

* Population with small sample size

Table S15. *Plectropomus leopardus* pairwise F_{ST} values based on nine microsatellite loci. Benjamini–Hochberg adjusted *P* < 0.01, italics; Benjamini–Hochberg adjusted P < 0.001, bold and italics.

	Fiji	New Caledonia	Swain Reef	Townsville	Lizard Island	Hick's Reef	Taiwan	Palau	Palawan	Cebu	Scott Reef
New Caledonia	***0.171***										
Swain Reef	***0.204***	***0.058***									
Townsville	***0.095***	***0.071***	0.011								
Lizard Island	***0.110***	***0.063***	***0.028***	***0.026***							
Hick's Reef	***0.079***	***0.079***	-0.001	-0.001	***0.025***						
Taiwan	***0.124***	***0.271***	***0.229***	***0.194***	***0.199***	***0.191***					
Palau	***0.071***	***0.189***	***0.195***	***0.115***	***0.133***	***0.112***	***0.040***				
Palawan	***0.082***	***0.166***	***0.173***	***0.082***	***0.106***	***0.064***	0.021	-0.018			
Cebu	***0.087***	***0.207***	***0.187***	***0.133***	***0.168***	***0.138***	***0.166***	***0.113***	***0.047***		
Scott Reef	***0.172***	***0.206***	***0.217***	***0.115***	***0.156***	***0.128***	***0.180***	***0.140***	***0.123***	***0.180***	
Abrolhos Islands	***0.065***	***0.187***	***0.143***	***0.098***	***0.103***	***0.104***	***0.098***	***0.076***	***0.032***	***0.124***	***0.043***

Table S16. Results of linkage disequilibrium test. Uncorrected p-value < 0.01 are highlighted in yellow.

Epinephelus polyphekadion

Pop	Locus#1	Locus#2	*P*
FJ	EP11	EP28	0.0016
FJ	EP11	EP25	0.7911
FJ	EP28	EP25	0.0703
FJ	EP11	EP33	0.6989
FJ	EP28	EP33	0.2376
FJ	EP25	EP33	0.0000
FJ	EP11	EP09	0.6081
FJ	EP28	EP09	0.3782
FJ	EP25	EP09	0.4131
FJ	EP33	EP09	0.7647
FJ	EP11	EP14	0.0161
FJ	EP28	EP14	0.1720
FJ	EP25	EP14	0.2697
FJ	EP33	EP14	0.0151
FJ	EP09	EP14	0.1248
FJ	EP11	EP35	0.4539
FJ	EP28	EP35	0.3847
FJ	EP25	EP35	0.7894
FJ	EP33	EP35	1.0000
FJ	EP09	EP35	0.2711
FJ	EP14	EP35	1.0000
FJ	EP11	EP34	0.5643
FJ	EP28	EP34	0.8440
FJ	EP25	EP34	0.0770
FJ	EP33	EP34	0.0062
FJ	EP09	EP34	0.2901
FJ	EP14	EP34	0.0154
FJ	EP35	EP34	1.0000
FJ	EP11	EP02	1.0000
FJ	EP28	EP02	0.8033
FJ	EP25	EP02	0.5314
FJ	EP33	EP02	0.5581
FJ	EP09	EP02	1.0000
FJ	EP14	EP02	0.2075
FJ	EP35	EP02	0.3220
FJ	EP34	EP02	0.5158
FJ	EP11	EP21	0.8480
FJ	EP28	EP21	0.4222
FJ	EP25	EP21	0.0138
FJ	EP33	EP21	0.0804
FJ	EP09	EP21	0.5364
FJ	EP14	EP21	0.2467
FJ	EP35	EP21	0.5677
FJ	EP34	EP21	0.3426
FJ	EP02	EP21	0.3641
FJ	EP11	EP20	0.8256
FJ	EP28	EP20	0.4006
FJ	EP25	EP20	0.5203
FJ	EP33	EP20	0.8443
FJ	EP09	EP20	0.2795
FJ	EP14	EP20	0.5950
FJ	EP35	EP20	1.0000
FJ	EP34	EP20	0.2203
FJ	EP02	EP20	0.0747
FJ	EP21	EP20	0.7129
WA	EP11	EP28	NA
WA	EP11	EP25	0.2987
WA	EP28	EP25	NA
WA	EP11	EP33	0.8683
WA	EP28	EP33	NA
WA	EP25	EP33	1.0000
WA	EP11	EP09	1.0000
WA	EP28	EP09	NA
WA	EP25	EP09	0.7616
WA	EP33	EP09	0.0448
WA	EP11	EP14	0.7128
WA	EP28	EP14	NA
WA	EP25	EP14	1.0000
WA	EP33	EP14	1.0000
WA	EP09	EP14	0.9266
WA	EP11	EP35	1.0000
WA	EP28	EP35	NA
WA	EP25	EP35	NA
WA	EP33	EP35	NA
WA	EP09	EP35	0.3331
WA	EP14	EP35	NA
WA	EP11	EP34	0.5499
WA	EP28	EP34	NA
WA	EP25	EP34	1.0000
WA	EP33	EP34	1.0000
WA	EP09	EP34	0.6661
WA	EP14	EP34	1.0000
WA	EP35	EP34	NA

Plectropomus areolatus

Pop	Locus#1	Locus#2	*P*
UM	Pm5	Pm3	0.3465
UM	Pm5	PaD2	0.5041
UM	Pm3	PaD2	0.7785
UM	Pm5	Pb111	0.5631
UM	Pm3	Pb111	0.5748
UM	PaD2	Pb111	0.6653
UM	Pm5	Pb120	0.8062
UM	Pm3	Pb120	0.4011
UM	PaD2	Pb120	0.5746
UM	Pb111	Pb120	0.1991
UM	Pm5	Pa114	0.3862
UM	Pm3	Pa114	0.9892
UM	PaD2	Pa114	0.7482
UM	Pb111	Pa114	0.0726
UM	Pb120	Pa114	0.0328
UM	Pm5	Pa117	0.2277
UM	Pm3	Pa117	0.9614
UM	PaD2	Pa117	0.4705
UM	Pb111	Pa117	0.0452
UM	Pb120	Pa117	0.0035
UM	Pa114	Pa117	0.3263
UM	Pm5	Pm6	0.7359
UM	Pm3	Pm6	0.3947
UM	PaD2	Pm6	0.0418
UM	Pb111	Pm6	0.0512
UM	Pb120	Pm6	0.5226
UM	Pa114	Pm6	0.7275
UM	Pa117	Pm6	0.2881
UM	Pm5	Pma038	0.8511
UM	Pm3	Pma038	0.9150
UM	PaD2	Pma038	0.3175
UM	Pb111	Pma038	0.6242
UM	Pb120	Pma038	1.0000
UM	Pa114	Pma038	0.2708
UM	Pa117	Pma038	1.0000
UM	Pm6	Pma038	1.0000
TU	Pm5	Pm3	0.5269
TU	Pm5	PaD2	0.3895
TU	Pm3	PaD2	0.1885
TU	Pm5	Pb111	0.0653
TU	Pm3	Pb111	0.9028
TU	PaD2	Pb111	0.1809
TU	Pm5	Pb120	0.1228
TU	Pm3	Pb120	0.0047
TU	PaD2	Pb120	0.1994
TU	Pb111	Pb120	0.4091
TU	Pm5	Pa114	0.7431
TU	Pm3	Pa114	0.1974
TU	PaD2	Pa114	0.0459
TU	Pb111	Pa114	0.0413
TU	Pb120	Pa114	0.2204
TU	Pm5	Pa117	0.0018
TU	Pm3	Pa117	0.0381
TU	PaD2	Pa117	0.7291
TU	Pb111	Pa117	0.8245
TU	Pb120	Pa117	0.0014
TU	Pa114	Pa117	0.5469
TU	Pm5	Pm6	0.0857
TU	Pm3	Pm6	0.1649
TU	PaD2	Pm6	0.1544
TU	Pb111	Pm6	0.3275
TU	Pb120	Pm6	0.3341
TU	Pa114	Pm6	0.6258
TU	Pa117	Pm6	0.3997
TU	Pm5	Pma038	0.2758
TU	Pm3	Pma038	0.0198
TU	PaD2	Pma038	0.2559
TU	Pb111	Pma038	0.2536
TU	Pb120	Pma038	0.0066
TU	Pa114	Pma038	0.2549
TU	Pa117	Pma038	0.0014
TU	Pm6	Pma038	0.3193
JI	Pm5	Pm3	0.1262
JI	Pm5	PaD2	0.3924
JI	Pm3	PaD2	0.0649
JI	Pm5	Pb111	0.7180
JI	Pm3	Pb111	0.8013
JI	PaD2	Pb111	0.4771
JI	Pm5	Pb120	0.6048
JI	Pm3	Pb120	0.3838
JI	PaD2	Pb120	0.8793
JI	Pb111	Pb120	0.0882
JI	Pm5	Pa114	0.2896

Plectropomus leopardus

Pop	Locus#1	Locus#2	*P*
AR	Pma101	Pma109	0.1672
AR	Pma101	Pma106	0.1684
AR	Pma109	Pma106	0.0577
AR	Pma101	Ple002	0.9966
AR	Pma109	Ple002	0.9990
AR	Pma106	Ple002	1.0000
AR	Pma101	Pma090	0.7406
AR	Pma109	Pma090	0.5925
AR	Pma106	Pma090	0.9116
AR	Ple002	Pma090	0.3841
AR	Pma101	Pma112	0.7358
AR	Pma109	Pma112	0.4616
AR	Pma106	Pma112	1.0000
AR	Ple002	Pma112	0.6971
AR	Pma090	Pma112	0.3158
AR	Pma101	Pm3	0.0245
AR	Pma109	Pm3	0.0165
AR	Pma106	Pm3	0.8561
AR	Ple002	Pm3	0.5041
AR	Pma090	Pm3	0.8222
AR	Pma112	Pm3	0.1558
AR	Pma101	Pma022	0.4055
AR	Pma109	Pma022	0.3490
AR	Pma106	Pma022	0.6018
AR	Ple002	Pma022	0.2469
AR	Pma090	Pma022	0.9084
AR	Pma112	Pma022	1.0000
AR	Pm3	Pma022	0.0511
AR	Pma101	Ple005	0.7422
AR	Pma109	Ple005	0.6001
AR	Pma106	Ple005	0.6382
AR	Ple002	Ple005	0.6411
AR	Pma090	Ple005	0.8600
AR	Pma112	Ple005	1.0000
AR	Pm3	Ple005	0.6911
AR	Pma022	Ple005	0.8297
SR	Pma101	Pma109	0.2580
SR	Pma101	Pma106	NA
SR	Pma109	Pma106	0.0540
SR	Pma101	Ple002	0.3584
SR	Pma109	Ple002	0.0178
SR	Pma106	Ple002	0.2541
SR	Pma101	Pma090	0.3174
SR	Pma109	Pma090	0.2767
SR	Pma106	Pma090	0.1652
SR	Ple002	Pma090	0.1630
SR	Pma101	Pma112	0.0127
SR	Pma109	Pma112	0.2746
SR	Pma106	Pma112	0.0453
SR	Ple002	Pma112	0.1023
SR	Pma090	Pma112	0.0248
SR	Pma101	Pm3	0.7327
SR	Pma109	Pm3	0.8717
SR	Pma106	Pm3	NA
SR	Ple002	Pm3	1.0000
SR	Pma090	Pm3	0.6776
SR	Pma112	Pm3	0.5709
SR	Pma101	Pma022	0.5254
SR	Pma109	Pma022	0.7779
SR	Pma106	Pma022	0.1919
SR	Ple002	Pma022	0.5235
SR	Pma090	Pma022	0.1041
SR	Pma112	Pma022	0.0424
SR	Pm3	Pma022	1.0000
SR	Pma101	Ple005	NA
SR	Pma109	Ple005	0.1031
SR	Pma106	Ple005	0.1705
SR	Ple002	Ple005	0.2638
SR	Pma090	Ple005	0.1789
SR	Pma112	Ple005	0.3209
SR	Pm3	Ple005	1.0000
SR	Pma022	Ple005	0.5084
PL	Pma101	Pma109	0.8099
PL	Pma101	Pma106	0.5803
PL	Pma109	Pma106	0.1798
PL	Pma101	Ple002	0.1149
PL	Pma109	Ple002	0.3781
PL	Pma106	Ple002	0.0416
PL	Pma101	Pma090	0.3261
PL	Pma109	Pma090	0.1883
PL	Pma106	Pma090	0.5320
PL	Ple002	Pma090	0.6153
PL	Pma101	Pma112	0.3067

Epinephelus polyphekadion

Pop	Locus#1	Locus#2	*P*
WA	EP11	EP02	1.0000
WA	EP28	EP02	NA
WA	EP25	EP02	0.8988
WA	EP33	EP02	0.4773
WA	EP09	EP02	0.9829
WA	EP14	EP02	0.5315
WA	EP35	EP02	NA
WA	EP34	EP02	0.3109
WA	EP11	EP21	0.3076
WA	EP28	EP21	NA
WA	EP25	EP21	0.3987
WA	EP33	EP21	1.0000
WA	EP09	EP21	0.8696
WA	EP14	EP21	1.0000
WA	EP35	EP21	NA
WA	EP34	EP21	0.4356
WA	EP02	EP21	0.9085
WA	EP11	EP20	0.3996
WA	EP28	EP20	NA
WA	EP25	EP20	0.6745
WA	EP33	EP20	1.0000
WA	EP09	EP20	0.2430
WA	EP14	EP20	1.0000
WA	EP35	EP20	NA
WA	EP34	EP20	1.0000
WA	EP02	EP20	0.8990
WA	EP21	EP20	1.0000
ML	EP11	EP28	0.0468
ML	EP11	EP25	0.0574
ML	EP28	EP25	0.2950
ML	EP11	EP33	0.1837
ML	EP28	EP33	0.1711
ML	EP25	EP33	0.1233
ML	EP11	EP09	0.2996
ML	EP28	EP09	0.2442
ML	EP25	EP09	0.2799
ML	EP33	EP09	0.2275
ML	EP11	EP14	0.8535
ML	EP28	EP14	0.0961
ML	EP25	EP14	0.3901
ML	EP33	EP14	0.0032
ML	EP09	EP14	0.0549
ML	EP11	EP35	0.1742
ML	EP28	EP35	0.1927
ML	EP25	EP35	0.1204
ML	EP33	EP35	0.8430
ML	EP09	EP35	0.1769
ML	EP14	EP35	0.5165
ML	EP11	EP34	0.0662
ML	EP28	EP34	0.1754
ML	EP25	EP34	0.3597
ML	EP33	EP34	0.0246
ML	EP09	EP34	0.0896
ML	EP14	EP34	0.3595
ML	EP35	EP34	0.0405
ML	EP11	EP02	0.9010
ML	EP28	EP02	0.6699
ML	EP25	EP02	0.0349
ML	EP33	EP02	0.4639
ML	EP09	EP02	0.0073
ML	EP14	EP02	0.8162
ML	EP35	EP02	0.2432
ML	EP34	EP02	0.6912
ML	EP11	EP21	0.4079
ML	EP28	EP21	0.8577
ML	EP25	EP21	0.1946
ML	EP33	EP21	0.8370
ML	EP09	EP21	0.0115
ML	EP14	EP21	0.8338
ML	EP35	EP21	0.5469
ML	EP34	EP21	0.3621
ML	EP02	EP21	0.0685
ML	EP11	EP20	1.0000
ML	EP28	EP20	0.8783
ML	EP25	EP20	0.7679
ML	EP33	EP20	0.4890
ML	EP09	EP20	0.8217
ML	EP14	EP20	0.1017
ML	EP35	EP20	0.5393
ML	EP34	EP20	0.3313
ML	EP02	EP20	1.0000
ML	EP21	EP20	0.1694
NC	EP11	EP28	0.3797
NC	EP11	EP25	0.6717
NC	EP28	EP25	0.1399
NC	EP11	EP33	0.2594

Plectropomus areolatus

Pop	Locus#1	Locus#2	*P*
JI	Pm3	Pa114	0.9264
JI	PaD2	Pa114	0.4816
JI	Pb111	Pa114	0.9076
JI	Pb120	Pa114	0.0930
JI	Pm5	Pa117	0.3045
JI	Pm3	Pa117	0.8722
JI	PaD2	Pa117	0.7410
JI	Pb111	Pa117	0.6548
JI	Pb120	Pa117	0.7733
JI	Pa114	Pa117	0.7549
JI	Pm5	Pm6	0.6507
JI	Pm3	Pm6	0.2662
JI	PaD2	Pm6	0.4416
JI	Pb111	Pm6	0.6237
JI	Pb120	Pm6	0.1226
JI	Pa114	Pm6	0.9972
JI	Pa117	Pm6	0.6665
JI	Pm5	Pma038	0.3741
JI	Pm3	Pma038	1.0000
JI	PaD2	Pma038	0.2646
JI	Pb111	Pma038	0.8685
JI	Pb120	Pma038	1.0000
JI	Pa114	Pma038	0.5166
JI	Pa117	Pma038	1.0000
JI	Pm6	Pma038	1.0000
ML	Pm5	Pm3	0.8221
ML	Pm5	PaD2	NA
ML	Pm3	PaD2	NA
ML	Pm5	Pb111	NA
ML	Pm3	Pb111	NA
ML	PaD2	Pb111	NA
ML	Pm5	Pb120	NA
ML	Pm3	Pb120	NA
ML	PaD2	Pb120	NA
ML	Pb111	Pb120	NA
ML	Pm5	Pa114	0.2564
ML	Pm3	Pa114	0.0224
ML	PaD2	Pa114	NA
ML	Pb111	Pa114	NA
ML	Pb120	Pa114	NA
ML	Pm5	Pa117	1.0000
ML	Pm3	Pa117	1.0000
ML	PaD2	Pa117	NA
ML	Pb111	Pa117	NA
ML	Pb120	Pa117	NA
ML	Pa114	Pa117	0.6671
ML	Pm5	Pm6	0.2562
ML	Pm3	Pm6	0.0108
ML	PaD2	Pm6	NA
ML	Pb111	Pm6	NA
ML	Pb120	Pm6	NA
ML	Pa114	Pm6	0.2807
ML	Pa117	Pm6	1.0000
ML	Pm5	Pma038	0.3698
ML	Pm3	Pma038	0.5490
ML	PaD2	Pma038	NA
ML	Pb111	Pma038	NA
ML	Pb120	Pma038	NA
ML	Pa114	Pma038	0.1955
ML	Pa117	Pma038	1.0000
ML	Pm6	Pma038	0.4417
CK	Pm5	Pm3	0.0520
CK	Pm5	PaD2	0.4012
CK	Pm3	PaD2	0.3062
CK	Pm5	Pb111	0.1647
CK	Pm3	Pb111	0.7320
CK	PaD2	Pb111	0.1174
CK	Pm5	Pb120	0.9425
CK	Pm3	Pb120	0.4979
CK	PaD2	Pb120	0.0599
CK	Pb111	Pb120	0.3327
CK	Pm5	Pa114	0.6101
CK	Pm3	Pa114	0.5122
CK	PaD2	Pa114	0.2450
CK	Pb111	Pa114	0.8079
CK	Pb120	Pa114	0.0427
CK	Pm5	Pa117	0.4290
CK	Pm3	Pa117	0.6043
CK	PaD2	Pa117	0.9433
CK	Pb111	Pa117	0.7749
CK	Pb120	Pa117	0.2307
CK	Pa114	Pa117	0.1217
CK	Pm5	Pm6	0.7952
CK	Pm3	Pm6	0.8245
CK	PaD2	Pm6	0.9103
CK	Pb111	Pm6	0.5970

Plectropomus leopardus

Pop	Locus#1	Locus#2	*P*
PL	Pma109	Pma112	0.7531
PL	Pma106	Pma112	0.9226
PL	Ple002	Pma112	0.5545
PL	Pma090	Pma112	0.8836
PL	Pma101	Pm3	0.9608
PL	Pma109	Pm3	0.1822
PL	Pma106	Pm3	1.0000
PL	Ple002	Pm3	1.0000
PL	Pma090	Pm3	0.4523
PL	Pma112	Pm3	0.2881
PL	Pma101	Pma022	0.7887
PL	Pma109	Pma022	0.2127
PL	Pma106	Pma022	0.4177
PL	Ple002	Pma022	1.0000
PL	Pma090	Pma022	0.1390
PL	Pma112	Pma022	1.0000
PL	Pm3	Pma022	1.0000
PL	Pma101	Ple005	0.7759
PL	Pma109	Ple005	0.3863
PL	Pma106	Ple005	0.8164
PL	Ple002	Ple005	0.3782
PL	Pma090	Ple005	0.6041
PL	Pma112	Ple005	0.9416
PL	Pm3	Ple005	0.0683
PL	Pma022	Ple005	0.0935
PC	Pma101	Pma109	1.0000
PC	Pma101	Pma106	NA
PC	Pma109	Pma106	NA
PC	Pma101	Ple002	NA
PC	Pma109	Ple002	NA
PC	Pma106	Ple002	NA
PC	Pma101	Pma090	1.0000
PC	Pma109	Pma090	NA
PC	Pma106	Pma090	NA
PC	Ple002	Pma090	0.1545
PC	Pma101	Pma112	0.4848
PC	Pma109	Pma112	0.0976
PC	Pma106	Pma112	NA
PC	Ple002	Pma112	1.0000
PC	Pma090	Pma112	0.0445
PC	Pma101	Pm3	NA
PC	Pma109	Pm3	NA
PC	Pma106	Pm3	NA
PC	Ple002	Pm3	NA
PC	Pma090	Pm3	NA
PC	Pma112	Pm3	NA
PC	Pma101	Pma022	1.0000
PC	Pma109	Pma022	NA
PC	Pma106	Pma022	NA
PC	Ple002	Pma022	1.0000
PC	Pma090	Pma022	1.0000
PC	Pma112	Pma022	0.2346
PC	Pm3	Pma022	NA
PC	Pma101	Ple005	1.0000
PC	Pma109	Ple005	NA
PC	Pma106	Ple005	NA
PC	Ple002	Ple005	1.0000
PC	Pma090	Ple005	1.0000
PC	Pma112	Ple005	1.0000
PC	Pm3	Ple005	NA
PC	Pma022	Ple005	0.0376
PP	Pma101	Pma109	0.1070
PP	Pma101	Pma106	1.0000
PP	Pma109	Pma106	0.2564
PP	Pma101	Ple002	1.0000
PP	Pma109	Ple002	1.0000
PP	Pma106	Ple002	1.0000
PP	Pma101	Pma090	0.6856
PP	Pma109	Pma090	0.2333
PP	Pma106	Pma090	0.5006
PP	Ple002	Pma090	0.4001
PP	Pma101	Pma112	1.0000
PP	Pma109	Pma112	0.4216
PP	Pma106	Pma112	1.0000
PP	Ple002	Pma112	1.0000
PP	Pma090	Pma112	0.3863
PP	Pma101	Pm3	NA
PP	Pma109	Pm3	NA
PP	Pma106	Pm3	NA
PP	Ple002	Pm3	NA
PP	Pma090	Pm3	NA
PP	Pma112	Pm3	NA
PP	Pma101	Pma022	1.0000
PP	Pma109	Pma022	0.1388
PP	Pma106	Pma022	0.1242
PP	Ple002	Pma022	1.0000

Epinephelus polyphekadion			
**Pop**	**Locus#1**	**Locus#2**	***P***
NC	EP28	EP33	0.5095
NC	EP25	EP33	0.2518
NC	EP11	EP09	0.8658
NC	EP28	EP09	0.3943
NC	EP25	EP09	0.1151
NC	EP33	EP09	0.9920
NC	EP11	EP14	0.9307
NC	EP28	EP14	0.2566
NC	EP25	EP14	0.3951
NC	EP33	EP14	0.0536
NC	EP09	EP14	0.3705
NC	EP11	EP35	0.4369
NC	EP28	EP35	0.7892
NC	EP25	EP35	0.2993
NC	EP33	EP35	1.0000
NC	EP09	EP35	0.1301
NC	EP14	EP35	0.3717
NC	EP11	EP34	0.1094
NC	EP28	EP34	0.0019
NC	EP25	EP34	0.1097
NC	EP33	EP34	1.0000
NC	EP09	EP34	0.0336
NC	EP14	EP34	0.4892
NC	EP35	EP34	0.0553
NC	EP11	EP02	0.7831
NC	EP28	EP02	0.7451
NC	EP25	EP02	0.6089
NC	EP33	EP02	0.4769
NC	EP09	EP02	0.5524
NC	EP14	EP02	0.0916
NC	EP35	EP02	0.0001
NC	EP34	EP02	0.0892
NC	EP11	EP21	0.6402
NC	EP28	EP21	0.8697
NC	EP25	EP21	0.0166
NC	EP33	EP21	0.8449
NC	EP09	EP21	0.6671
NC	EP14	EP21	0.0298
NC	EP35	EP21	0.0337
NC	EP34	EP21	0.0389
NC	EP02	EP21	0.5014
NC	EP11	EP20	0.1931
NC	EP28	EP20	0.0750
NC	EP25	EP20	0.0088
NC	EP33	EP20	0.8503
NC	EP09	EP20	0.2995
NC	EP14	EP20	0.3124
NC	EP35	EP20	0.4315
NC	EP34	EP20	0.0275
NC	EP02	EP20	0.4173
NC	EP21	EP20	0.2300
PH	EP11	EP28	NA
PH	EP11	EP25	NA
PH	EP28	EP25	0.4335
PH	EP11	EP33	NA
PH	EP28	EP33	0.3989
PH	EP25	EP33	1.0000
PH	EP11	EP09	NA
PH	EP28	EP09	1.0000
PH	EP25	EP09	0.7884
PH	EP33	EP09	0.6486
PH	EP11	EP14	NA
PH	EP28	EP14	0.2503
PH	EP25	EP14	0.9959
PH	EP33	EP14	0.1114
PH	EP09	EP14	0.2391
PH	EP11	EP35	NA
PH	EP28	EP35	0.5593
PH	EP25	EP35	1.0000
PH	EP33	EP35	1.0000
PH	EP09	EP35	1.0000
PH	EP14	EP35	1.0000
PH	EP11	EP34	NA
PH	EP28	EP34	0.6258
PH	EP25	EP34	1.0000
PH	EP33	EP34	1.0000
PH	EP09	EP34	0.3052
PH	EP14	EP34	NA
PH	EP35	EP34	1.0000
PH	EP11	EP02	NA
PH	EP28	EP02	0.5787
PH	EP25	EP02	0.7768
PH	EP33	EP02	1.0000
PH	EP09	EP02	0.3942
PH	EP14	EP02	0.7808
PH	EP35	EP02	0.1503

Plectropomus areolatus			
**Pop**	**Locus#1**	**Locus#2**	***P***
CK	Pb120	Pm6	0.2815
CK	Pa114	Pm6	0.3016
CK	Pa117	Pm6	0.3036
CK	Pm5	Pma038	0.4089
CK	Pm3	Pma038	0.0534
CK	PaD2	Pma038	0.0474
CK	Pb111	Pma038	0.7500
CK	Pb120	Pma038	0.2983
CK	Pa114	Pma038	0.0597
CK	Pa117	Pma038	0.8439
CK	Pm6	Pma038	0.3317
CR	Pm5	Pm3	0.9433
CR	Pm5	PaD2	0.7877
CR	Pm3	PaD2	0.0878
CR	Pm5	Pb111	0.4078
CR	Pm3	Pb111	0.8641
CR	PaD2	Pb111	0.7689
CR	Pm5	Pb120	0.1563
CR	Pm3	Pb120	0.0855
CR	PaD2	Pb120	0.0191
CR	Pb111	Pb120	0.0440
CR	Pm5	Pa114	0.4558
CR	Pm3	Pa114	0.8074
CR	PaD2	Pa114	0.3488
CR	Pb111	Pa114	0.2755
CR	Pb120	Pa114	0.7946
CR	Pm5	Pa117	0.3373
CR	Pm3	Pa117	0.8674
CR	PaD2	Pa117	0.8316
CR	Pb111	Pa117	0.2989
CR	Pb120	Pa117	0.1642
CR	Pa114	Pa117	0.8032
CR	Pm5	Pm6	0.2786
CR	Pm3	Pm6	0.8508
CR	PaD2	Pm6	0.3864
CR	Pb111	Pm6	1.0000
CR	Pb120	Pm6	0.8800
CR	Pa114	Pm6	0.7952
CR	Pa117	Pm6	0.5733
CR	Pm5	Pma038	0.9910
CR	Pm3	Pma038	0.3913
CR	PaD2	Pma038	0.7965
CR	Pb111	Pma038	0.9556
CR	Pb120	Pma038	0.8042
CR	Pa114	Pma038	0.2274
CR	Pa117	Pma038	0.5862
CR	Pm6	Pma038	0.6646
SR	Pm5	Pm3	0.0768
SR	Pm5	PaD2	0.7219
SR	Pm3	PaD2	0.8516
SR	Pm5	Pb111	0.0765
SR	Pm3	Pb111	0.1433
SR	PaD2	Pb111	0.8638
SR	Pm5	Pb120	0.9651
SR	Pm3	Pb120	1.0000
SR	PaD2	Pb120	0.4518
SR	Pb111	Pb120	0.3819
SR	Pm5	Pa114	0.8501
SR	Pm3	Pa114	0.3892
SR	PaD2	Pa114	0.9457
SR	Pb111	Pa114	0.7801
SR	Pb120	Pa114	1.0000
SR	Pm5	Pa117	0.4618
SR	Pm3	Pa117	1.0000
SR	PaD2	Pa117	0.4261
SR	Pb111	Pa117	1.0000
SR	Pb120	Pa117	0.7630
SR	Pa114	Pa117	0.8540
SR	Pm5	Pm6	1.0000
SR	Pm3	Pm6	1.0000
SR	PaD2	Pm6	0.2467
SR	Pb111	Pm6	1.0000
SR	Pb120	Pm6	0.5084
SR	Pa114	Pm6	1.0000
SR	Pa117	Pm6	0.1314
SR	Pm5	Pma038	0.7364
SR	Pm3	Pma038	0.2717
SR	PaD2	Pma038	0.7954
SR	Pb111	Pma038	0.4556
SR	Pb120	Pma038	0.1751
SR	Pa114	Pma038	0.2995
SR	Pa117	Pma038	1.0000
SR	Pm6	Pma038	0.8977
IN	Pm5	Pm3	1.0000
IN	Pm5	PaD2	0.6114
IN	Pm3	PaD2	0.8406

Plectropomus leopardus			
**Pop**	**Locus#1**	**Locus#2**	***P***
PP	Pma090	Pma022	0.8166
PP	Pma112	Pma022	1.0000
PP	Pm3	Pma022	NA
PP	Pma101	Ple005	1.0000
PP	Pma109	Ple005	0.9066
PP	Pma106	Ple005	0.5126
PP	Ple002	Ple005	NA
PP	Pma090	Ple005	0.7498
PP	Pma112	Ple005	0.7160
PP	Pm3	Ple005	NA
PP	Pma022	Ple005	1.0000
TW	Pma101	Pma109	0.1417
TW	Pma101	Pma106	0.6386
TW	Pma109	Pma106	0.3575
TW	Pma101	Ple002	0.2110
TW	Pma109	Ple002	0.6530
TW	Pma106	Ple002	0.3282
TW	Pma101	Pma090	0.7077
TW	Pma109	Pma090	0.0331
TW	Pma106	Pma090	0.0394
TW	Ple002	Pma090	0.0102
TW	Pma101	Pma112	0.2701
TW	Pma109	Pma112	0.3250
TW	Pma106	Pma112	0.0162
TW	Ple002	Pma112	0.2960
TW	Pma090	Pma112	0.3635
TW	Pma101	Pm3	0.2987
TW	Pma109	Pm3	0.9740
TW	Pma106	Pm3	0.5378
TW	Ple002	Pm3	0.1373
TW	Pma090	Pm3	0.5442
TW	Pma112	Pm3	0.3966
TW	Pma101	Pma022	0.0025
TW	Pma109	Pma022	0.6918
TW	Pma106	Pma022	0.5663
TW	Ple002	Pma022	0.0784
TW	Pma090	Pma022	0.1169
TW	Pma112	Pma022	0.2766
TW	Pm3	Pma022	0.9689
TW	Pma101	Ple005	0.0010
TW	Pma109	Ple005	0.4433
TW	Pma106	Ple005	0.7581
TW	Ple002	Ple005	0.0217
TW	Pma090	Ple005	0.1777
TW	Pma112	Ple005	0.1522
TW	Pm3	Ple005	0.2986
TW	Pma022	Ple005	0.0641
LI	Pma101	Pma109	0.0974
LI	Pma101	Pma106	0.2614
LI	Pma109	Pma106	1.0000
LI	Pma101	Ple002	0.9511
LI	Pma109	Ple002	1.0000
LI	Pma106	Ple002	0.4959
LI	Pma101	Pma090	0.0463
LI	Pma109	Pma090	0.5578
LI	Pma106	Pma090	0.6596
LI	Ple002	Pma090	0.7435
LI	Pma101	Pma112	0.3431
LI	Pma109	Pma112	0.6048
LI	Pma106	Pma112	1.0000
LI	Ple002	Pma112	0.7179
LI	Pma090	Pma112	0.4789
LI	Pma101	Pm3	0.9014
LI	Pma109	Pm3	1.0000
LI	Pma106	Pm3	1.0000
LI	Ple002	Pm3	0.2787
LI	Pma090	Pm3	0.1533
LI	Pma112	Pm3	0.2084
LI	Pma101	Pma022	0.2317
LI	Pma109	Pma022	1.0000
LI	Pma106	Pma022	0.9027
LI	Ple002	Pma022	0.0633
LI	Pma090	Pma022	0.1125
LI	Pma112	Pma022	0.0463
LI	Pm3	Pma022	0.9041
LI	Pma101	Ple005	0.3994
LI	Pma109	Ple005	0.6738
LI	Pma106	Ple005	0.3266
LI	Ple002	Ple005	0.8438
LI	Pma090	Ple005	0.5967
LI	Pma112	Ple005	0.8171
LI	Pm3	Ple005	0.0846
LI	Pma022	Ple005	0.2335
HD	Pma101	Pma109	1.0000
HD	Pma101	Pma106	0.4487
HD	Pma109	Pma106	1.0000

Epinephelus polyphekadion			
**Pop**	**Locus#1**	**Locus#2**	***P***
PH	EP34	EP02	1.0000
PH	EP11	EP21	NA
PH	EP28	EP21	1.0000
PH	EP25	EP21	0.4411
PH	EP33	EP21	1.0000
PH	EP09	EP21	0.4634
PH	EP14	EP21	0.1476
PH	EP35	EP21	0.6135
PH	EP34	EP21	NA
PH	EP02	EP21	0.3681
PH	EP11	EP20	NA
PH	EP28	EP20	0.4164
PH	EP25	EP20	0.7929
PH	EP33	EP20	1.0000
PH	EP09	EP20	0.0260
PH	EP14	EP20	0.9129
PH	EP35	EP20	0.4694
PH	EP34	EP20	1.0000
PH	EP02	EP20	0.3155
PH	EP21	EP20	0.7232
PL	EP11	EP28	0.8974
PL	EP11	EP25	0.9919
PL	EP28	EP25	0.5714
PL	EP11	EP33	0.5816
PL	EP28	EP33	0.2217
PL	EP25	EP33	0.3148
PL	EP11	EP09	0.2752
PL	EP28	EP09	0.2692
PL	EP25	EP09	0.0857
PL	EP33	EP09	0.0942
PL	EP11	EP14	0.6229
PL	EP28	EP14	0.7259
PL	EP25	EP14	0.1156
PL	EP33	EP14	0.5431
PL	EP09	EP14	0.3505
PL	EP11	EP35	0.7814
PL	EP28	EP35	0.6743
PL	EP25	EP35	0.0046
PL	EP33	EP35	0.0044
PL	EP09	EP35	0.8167
PL	EP14	EP35	0.3679
PL	EP11	EP34	0.9623
PL	EP28	EP34	0.2023
PL	EP25	EP34	0.6095
PL	EP33	EP34	0.0064
PL	EP09	EP34	0.8677
PL	EP14	EP34	1.0000
PL	EP35	EP34	0.1311
PL	EP11	EP02	0.3824
PL	EP28	EP02	0.5421
PL	EP25	EP02	0.1341
PL	EP33	EP02	0.6845
PL	EP09	EP02	0.2294
PL	EP14	EP02	0.6072
PL	EP35	EP02	0.1330
PL	EP34	EP02	0.7828
PL	EP11	EP21	0.7733
PL	EP28	EP21	0.0352
PL	EP25	EP21	0.0887
PL	EP33	EP21	0.3385
PL	EP09	EP21	0.8768
PL	EP14	EP21	0.4420
PL	EP35	EP21	0.2014
PL	EP34	EP21	0.9417
PL	EP02	EP21	0.3989
PL	EP11	EP20	0.4900
PL	EP28	EP20	0.4818
PL	EP25	EP20	0.0158
PL	EP33	EP20	0.0726
PL	EP09	EP20	0.0959
PL	EP14	EP20	0.4141
PL	EP35	EP20	0.2131
PL	EP34	EP20	0.4096
PL	EP02	EP20	0.0501
PL	EP21	EP20	0.9401
PO	EP11	EP28	1.0000
PO	EP11	EP25	0.7208
PO	EP28	EP25	0.2721
PO	EP11	EP33	0.5833
PO	EP28	EP33	0.7228
PO	EP25	EP33	1.0000
PO	EP11	EP09	0.2400
PO	EP28	EP09	0.3899
PO	EP25	EP09	0.6845
PO	EP33	EP09	0.6586
PO	EP11	EP14	0.4154

Plectropomus areolatus			
**Pop**	**Locus#1**	**Locus#2**	***P***
IN	Pm5	Pb111	0.6421
IN	Pm3	Pb111	1.0000
IN	PaD2	Pb111	0.0349
IN	Pm5	Pb120	0.9333
IN	Pm3	Pb120	0.8337
IN	PaD2	Pb120	1.0000
IN	Pb111	Pb120	1.0000
IN	Pm5	Pa114	0.2535
IN	Pm3	Pa114	1.0000
IN	PaD2	Pa114	0.0683
IN	Pb111	Pa114	0.2198
IN	Pb120	Pa114	0.6004
IN	Pm5	Pa117	1.0000
IN	Pm3	Pa117	0.2938
IN	PaD2	Pa117	0.3344
IN	Pb111	Pa117	0.3611
IN	Pb120	Pa117	1.0000
IN	Pa114	Pa117	1.0000
IN	Pm5	Pm6	1.0000
IN	Pm3	Pm6	0.1077
IN	PaD2	Pm6	1.0000
IN	Pb111	Pm6	1.0000
IN	Pb120	Pm6	1.0000
IN	Pa114	Pm6	1.0000
IN	Pa117	Pm6	0.4910
IN	Pm5	Pma038	0.5032
IN	Pm3	Pma038	1.0000
IN	PaD2	Pma038	0.7953
IN	Pb111	Pma038	0.7522
IN	Pb120	Pma038	0.2662
IN	Pa114	Pma038	0.6294
IN	Pa117	Pma038	0.7825
IN	Pm6	Pma038	1.0000
PH	Pm5	Pm3	0.0396
PH	Pm5	PaD2	0.4784
PH	Pm3	PaD2	0.3230
PH	Pm5	Pb111	0.2167
PH	Pm3	Pb111	0.0010
PH	PaD2	Pb111	0.1902
PH	Pm5	Pb120	0.3384
PH	Pm3	Pb120	0.2015
PH	PaD2	Pb120	0.2415
PH	Pb111	Pb120	0.4334
PH	Pm5	Pa114	0.0379
PH	Pm3	Pa114	0.0013
PH	PaD2	Pa114	0.1174
PH	Pb111	Pa114	0.0019
PH	Pb120	Pa114	0.3268
PH	Pm5	Pa117	0.3218
PH	Pm3	Pa117	0.1488
PH	PaD2	Pa117	0.5609
PH	Pb111	Pa117	0.0326
PH	Pb120	Pa117	0.9672
PH	Pa114	Pa117	0.0034
PH	Pm5	Pm6	0.0181
PH	Pm3	Pm6	0.0288
PH	PaD2	Pm6	0.0497
PH	Pb111	Pm6	0.0000
PH	Pb120	Pm6	0.1924
PH	Pa114	Pm6	0.0001
PH	Pa117	Pm6	0.2629
PH	Pm5	Pma038	0.0614
PH	Pm3	Pma038	0.0131
PH	PaD2	Pma038	0.3465
PH	Pb111	Pma038	0.5871
PH	Pb120	Pma038	0.0710
PH	Pa114	Pma038	0.0349
PH	Pa117	Pma038	0.7409
PH	Pm6	Pma038	0.1612
PL	Pm5	Pm3	0.1757
PL	Pm5	PaD2	0.3922
PL	Pm3	PaD2	0.5749
PL	Pm5	Pb111	0.3600
PL	Pm3	Pb111	1.0000
PL	PaD2	Pb111	0.1343
PL	Pm5	Pb120	0.6100
PL	Pm3	Pb120	0.6623
PL	PaD2	Pb120	0.5995
PL	Pb111	Pb120	0.9167
PL	Pm5	Pa114	0.1847
PL	Pm3	Pa114	0.8742
PL	PaD2	Pa114	0.2994
PL	Pb111	Pa114	0.4367
PL	Pb120	Pa114	0.5135
PL	Pm5	Pa117	0.9697
PL	Pm3	Pa117	0.9262

Plectropomus leopardus			
**Pop**	**Locus#1**	**Locus#2**	***P***
HD	Pma101	Ple002	0.0901
HD	Pma109	Ple002	1.0000
HD	Pma106	Ple002	1.0000
HD	Pma101	Pma090	0.8481
HD	Pma109	Pma090	1.0000
HD	Pma106	Pma090	0.0541
HD	Ple002	Pma090	0.6318
HD	Pma101	Pma112	0.4232
HD	Pma109	Pma112	1.0000
HD	Pma106	Pma112	0.1344
HD	Ple002	Pma112	0.4011
HD	Pma090	Pma112	0.9308
HD	Pma101	Pm3	0.0336
HD	Pma109	Pm3	1.0000
HD	Pma106	Pm3	1.0000
HD	Ple002	Pm3	1.0000
HD	Pma090	Pm3	0.1934
HD	Pma112	Pm3	0.1091
HD	Pma101	Pma022	0.4052
HD	Pma109	Pma022	0.7249
HD	Pma106	Pma022	0.8490
HD	Ple002	Pma022	0.3411
HD	Pma090	Pma022	0.0410
HD	Pma112	Pma022	0.5226
HD	Pm3	Pma022	0.1438
HD	Pma101	Ple005	0.3395
HD	Pma109	Ple005	1.0000
HD	Pma106	Ple005	1.0000
HD	Ple002	Ple005	0.3516
HD	Pma090	Ple005	0.4905
HD	Pma112	Ple005	1.0000
HD	Pm3	Ple005	1.0000
HD	Pma022	Ple005	0.2205
TV	Pma101	Pma109	1.0000
TV	Pma101	Pma106	1.0000
TV	Pma109	Pma106	1.0000
TV	Pma101	Ple002	0.5762
TV	Pma109	Ple002	1.0000
TV	Pma106	Ple002	0.1290
TV	Pma101	Pma090	0.6965
TV	Pma109	Pma090	0.3499
TV	Pma106	Pma090	0.1725
TV	Ple002	Pma090	0.3473
TV	Pma101	Pma112	0.1245
TV	Pma109	Pma112	0.3779
TV	Pma106	Pma112	0.6306
TV	Ple002	Pma112	0.4997
TV	Pma090	Pma112	0.3442
TV	Pma101	Pm3	0.4413
TV	Pma109	Pm3	1.0000
TV	Pma106	Pm3	0.6905
TV	Ple002	Pm3	1.0000
TV	Pma090	Pm3	0.8197
TV	Pma112	Pm3	0.7786
TV	Pma101	Pma022	0.2896
TV	Pma109	Pma022	0.0562
TV	Pma106	Pma022	0.8501
TV	Ple002	Pma022	0.1535
TV	Pma090	Pma022	0.2889
TV	Pma112	Pma022	0.6845
TV	Pm3	Pma022	0.4714
TV	Pma101	Ple005	0.7555
TV	Pma109	Ple005	1.0000
TV	Pma106	Ple005	0.1468
TV	Ple002	Ple005	0.0034
TV	Pma090	Ple005	0.9556
TV	Pma112	Ple005	0.8417
TV	Pm3	Ple005	0.4269
TV	Pma022	Ple005	0.0041
SW	Pma101	Pma109	1.0000
SW	Pma101	Pma106	1.0000
SW	Pma109	Pma106	NA
SW	Pma101	Ple002	0.2792
SW	Pma109	Ple002	1.0000
SW	Pma106	Ple002	0.0587
SW	Pma101	Pma090	0.4892
SW	Pma109	Pma090	1.0000
SW	Pma106	Pma090	0.1525
SW	Ple002	Pma090	0.0131
SW	Pma101	Pma112	0.2332
SW	Pma109	Pma112	1.0000
SW	Pma106	Pma112	1.0000
SW	Ple002	Pma112	0.0467
SW	Pma090	Pma112	0.8571
SW	Pma101	Pm3	0.8482
SW	Pma109	Pm3	1.0000

Epinephelus polyphekadion			
**Pop**	**Locus#1**	**Locus#2**	***P***
PO	EP28	EP14	0.4212
PO	EP25	EP14	0.2307
PO	EP33	EP14	0.5804
PO	EP09	EP14	0.8291
PO	EP11	EP35	0.6271
PO	EP28	EP35	0.9008
PO	EP25	EP35	0.1470
PO	EP33	EP35	0.1254
PO	EP09	EP35	0.7163
PO	EP14	EP35	0.5152
PO	EP11	EP34	0.7178
PO	EP28	EP34	0.6707
PO	EP25	EP34	1.0000
PO	EP33	EP34	1.0000
PO	EP09	EP34	0.8300
PO	EP14	EP34	1.0000
PO	EP35	EP34	1.0000
PO	EP11	EP02	0.2084
PO	EP28	EP02	0.9369
PO	EP25	EP02	0.9900
PO	EP33	EP02	0.3356
PO	EP09	EP02	0.5774
PO	EP14	EP02	0.7728
PO	EP35	EP02	0.5084
PO	EP34	EP02	0.3559
PO	EP11	EP21	1.0000
PO	EP28	EP21	0.7978
PO	EP25	EP21	0.1170
PO	EP33	EP21	0.7048
PO	EP09	EP21	0.7236
PO	EP14	EP21	0.4493
PO	EP35	EP21	1.0000
PO	EP34	EP21	1.0000
PO	EP02	EP21	0.1065
PO	EP11	EP20	0.3592
PO	EP28	EP20	0.9602
PO	EP25	EP20	0.8794
PO	EP33	EP20	1.0000
PO	EP09	EP20	0.9747
PO	EP14	EP20	0.7975
PO	EP35	EP20	1.0000
PO	EP34	EP20	0.6759
PO	EP02	EP20	0.4525
PO	EP21	EP20	0.3089
TM	EP11	EP28	0.1175
TM	EP11	EP25	0.8527
TM	EP28	EP25	0.4696
TM	EP11	EP33	0.2798
TM	EP28	EP33	0.5303
TM	EP25	EP33	0.2370
TM	EP11	EP09	0.5586
TM	EP28	EP09	1.0000
TM	EP25	EP09	0.4092
TM	EP33	EP09	0.7811
TM	EP11	EP14	0.8817
TM	EP28	EP14	1.0000
TM	EP25	EP14	0.0498
TM	EP33	EP14	0.7005
TM	EP09	EP14	0.8726
TM	EP11	EP35	0.9270
TM	EP28	EP35	0.7066
TM	EP25	EP35	0.7918
TM	EP33	EP35	1.0000
TM	EP09	EP35	1.0000
TM	EP14	EP35	0.8160
TM	EP11	EP34	0.8456
TM	EP28	EP34	1.0000
TM	EP25	EP34	0.6588
TM	EP33	EP34	1.0000
TM	EP09	EP34	0.9363
TM	EP14	EP34	0.6336
TM	EP35	EP34	1.0000
TM	EP11	EP02	1.0000
TM	EP28	EP02	1.0000
TM	EP25	EP02	0.7062
TM	EP33	EP02	0.7647
TM	EP09	EP02	1.0000
TM	EP14	EP02	1.0000
TM	EP35	EP02	0.7060
TM	EP34	EP02	0.5300
TM	EP11	EP21	0.6393
TM	EP28	EP21	1.0000
TM	EP25	EP21	0.3078
TM	EP33	EP21	0.5028
TM	EP09	EP21	0.7124
TM	EP14	EP21	0.9603

Plectropomus areolatus			
**Pop**	**Locus#1**	**Locus#2**	***P***
PL	PaD2	Pa117	0.4086
PL	Pb111	Pa117	0.0511
PL	Pb120	Pa117	1.0000
PL	Pa114	Pa117	0.4826
PL	Pm5	Pm6	0.3307
PL	Pm3	Pm6	1.0000
PL	PaD2	Pm6	0.8477
PL	Pb111	Pm6	0.1735
PL	Pb120	Pm6	0.2159
PL	Pa114	Pm6	1.0000
PL	Pa117	Pm6	0.6495
PL	Pm5	Pma038	0.3755
PL	Pm3	Pma038	0.4834
PL	PaD2	Pma038	0.0785
PL	Pb111	Pma038	0.8023
PL	Pb120	Pma038	0.5590
PL	Pa114	Pma038	0.7667
PL	Pa117	Pma038	0.6228
PL	Pm6	Pma038	0.8122
PO	Pm5	Pm3	0.1719
PO	Pm5	PaD2	0.7879
PO	Pm3	PaD2	0.9016
PO	Pm5	Pb111	0.8497
PO	Pm3	Pb111	0.7288
PO	PaD2	Pb111	0.5806
PO	Pm5	Pb120	0.2048
PO	Pm3	Pb120	0.0731
PO	PaD2	Pb120	0.4346
PO	Pb111	Pb120	0.1669
PO	Pm5	Pa114	0.9901
PO	Pm3	Pa114	0.4811
PO	PaD2	Pa114	0.1105
PO	Pb111	Pa114	0.7251
PO	Pb120	Pa114	0.0967
PO	Pm5	Pa117	0.5333
PO	Pm3	Pa117	0.2384
PO	PaD2	Pa117	0.9414
PO	Pb111	Pa117	0.6437
PO	Pb120	Pa117	0.2586
PO	Pa114	Pa117	0.5398
PO	Pm5	Pm6	0.7180
PO	Pm3	Pm6	0.7070
PO	PaD2	Pm6	0.3360
PO	Pb111	Pm6	0.0658
PO	Pb120	Pm6	0.1105
PO	Pa114	Pm6	0.7756
PO	Pa117	Pm6	0.2015
PO	Pm5	Pma038	0.2795
PO	Pm3	Pma038	0.2429
PO	PaD2	Pma038	0.0559
PO	Pb111	Pma038	0.6709
PO	Pb120	Pma038	0.0570
PO	Pa114	Pma038	0.9879
PO	Pa117	Pma038	0.4406
PO	Pm6	Pma038	0.5081
TS	Pm5	Pm3	0.6334
TS	Pm5	PaD2	0.6504
TS	Pm3	PaD2	0.5548
TS	Pm5	Pb111	0.0398
TS	Pm3	Pb111	0.7769
TS	PaD2	Pb111	0.7768
TS	Pm5	Pb120	0.4247
TS	Pm3	Pb120	0.3076
TS	PaD2	Pb120	0.1106
TS	Pb111	Pb120	0.5962
TS	Pm5	Pa114	0.4452
TS	Pm3	Pa114	0.6644
TS	PaD2	Pa114	0.2914
TS	Pb111	Pa114	0.6278
TS	Pb120	Pa114	0.4957
TS	Pm5	Pa117	0.7812
TS	Pm3	Pa117	0.8555
TS	PaD2	Pa117	0.2745
TS	Pb111	Pa117	0.1155
TS	Pb120	Pa117	0.8514
TS	Pa114	Pa117	0.2152
TS	Pm5	Pm6	0.4147
TS	Pm3	Pm6	0.6809
TS	PaD2	Pm6	0.0372
TS	Pb111	Pm6	0.9710
TS	Pb120	Pm6	0.3056
TS	Pa114	Pm6	0.0344
TS	Pa117	Pm6	0.1963
TS	Pm5	Pma038	0.1608
TS	Pm3	Pma038	0.9618
TS	PaD2	Pma038	0.8526

Plectropomus leopardus			
**Pop**	**Locus#1**	**Locus#2**	***P***
SW	Pma106	Pm3	0.8027
SW	Ple002	Pm3	0.0523
SW	Pma090	Pm3	0.5287
SW	Pma112	Pm3	0.6556
SW	Pma101	Pma022	0.0182
SW	Pma109	Pma022	0.6813
SW	Pma106	Pma022	0.0432
SW	Ple002	Pma022	0.0011
SW	Pma090	Pma022	0.1946
SW	Pma112	Pma022	0.0212
SW	Pm3	Pma022	0.4454
SW	Pma101	Ple005	1.0000
SW	Pma109	Ple005	1.0000
SW	Pma106	Ple005	1.0000
SW	Ple002	Ple005	0.1280
SW	Pma090	Ple005	0.8116
SW	Pma112	Ple005	0.2013
SW	Pm3	Ple005	0.0751
SW	Pma022	Ple005	0.8191
NC	Pma101	Pma109	1.0000
NC	Pma101	Pma106	0.3730
NC	Pma109	Pma106	0.7240
NC	Pma101	Ple002	0.8850
NC	Pma109	Ple002	1.0000
NC	Pma106	Ple002	0.2752
NC	Pma101	Pma090	0.7273
NC	Pma109	Pma090	0.2454
NC	Pma106	Pma090	0.6750
NC	Ple002	Pma090	0.0950
NC	Pma101	Pma112	0.4020
NC	Pma109	Pma112	1.0000
NC	Pma106	Pma112	0.6728
NC	Ple002	Pma112	0.4877
NC	Pma090	Pma112	0.3430
NC	Pma101	Pm3	0.5286
NC	Pma109	Pm3	1.0000
NC	Pma106	Pm3	0.2606
NC	Ple002	Pm3	0.6103
NC	Pma090	Pm3	0.2498
NC	Pma112	Pm3	0.4054
NC	Pma101	Pma022	0.7411
NC	Pma109	Pma022	NA
NC	Pma106	Pma022	0.5733
NC	Ple002	Pma022	0.3600
NC	Pma090	Pma022	0.2897
NC	Pma112	Pma022	0.6571
NC	Pm3	Pma022	0.3886
NC	Pma101	Ple005	0.0745
NC	Pma109	Ple005	1.0000
NC	Pma106	Ple005	0.3550
NC	Ple002	Ple005	0.5002
NC	Pma090	Ple005	0.7031
NC	Pma112	Ple005	0.6789
NC	Pm3	Ple005	0.0202
NC	Pma022	Ple005	0.3152
FJ	Pma101	Pma109	1.0000
FJ	Pma101	Pma106	1.0000
FJ	Pma109	Pma106	0.1754
FJ	Pma101	Ple002	0.1282
FJ	Pma109	Ple002	1.0000
FJ	Pma106	Ple002	0.0157
FJ	Pma101	Pma090	0.3171
FJ	Pma109	Pma090	1.0000
FJ	Pma106	Pma090	0.9491
FJ	Ple002	Pma090	0.2295
FJ	Pma101	Pma112	1.0000
FJ	Pma109	Pma112	1.0000
FJ	Pma106	Pma112	0.2501
FJ	Ple002	Pma112	0.1175
FJ	Pma090	Pma112	1.0000
FJ	Pma101	Pm3	1.0000
FJ	Pma109	Pm3	0.1779
FJ	Pma106	Pm3	0.0370
FJ	Ple002	Pm3	0.1968
FJ	Pma090	Pm3	0.2754
FJ	Pma112	Pm3	0.3275
FJ	Pma101	Pma022	1.0000
FJ	Pma109	Pma022	1.0000
FJ	Pma106	Pma022	0.0681
FJ	Ple002	Pma022	0.2012
FJ	Pma090	Pma022	0.3998
FJ	Pma112	Pma022	1.0000
FJ	Pm3	Pma022	0.0319
FJ	Pma101	Ple005	0.3528
FJ	Pma109	Ple005	1.0000
FJ	Pma106	Ple005	1.0000

Epinephelus polyphekadion			
**Pop**	**Locus#1**	**Locus#2**	***P***
TM	EP35	EP21	0.9489
TM	EP34	EP21	0.1077
TM	EP02	EP21	0.5882
TM	EP11	EP20	0.3542
TM	EP28	EP20	0.7645
TM	EP25	EP20	0.8565
TM	EP33	EP20	1.0000
TM	EP09	EP20	0.0052
TM	EP14	EP20	0.8348
TM	EP35	EP20	1.0000
TM	EP34	EP20	0.4986
TM	EP02	EP20	0.2346
TM	EP21	EP20	0.8009
CK	EP11	EP28	1.0000
CK	EP11	EP25	0.1731
CK	EP28	EP25	0.7996
CK	EP11	EP33	0.1507
CK	EP28	EP33	0.0445
CK	EP25	EP33	0.9750
CK	EP11	EP09	0.0687
CK	EP28	EP09	0.4672
CK	EP25	EP09	0.0494
CK	EP33	EP09	0.0725
CK	EP11	EP14	0.5002
CK	EP28	EP14	0.5795
CK	EP25	EP14	0.9785
CK	EP33	EP14	0.7884
CK	EP09	EP14	0.3494
CK	EP11	EP35	0.6688
CK	EP28	EP35	0.5964
CK	EP25	EP35	0.6219
CK	EP33	EP35	1.0000
CK	EP09	EP35	0.0552
CK	EP14	EP35	0.0204
CK	EP11	EP34	0.8713
CK	EP28	EP34	0.4752
CK	EP25	EP34	0.7307
CK	EP33	EP34	0.1291
CK	EP09	EP34	0.5426
CK	EP14	EP34	1.0000
CK	EP35	EP34	1.0000
CK	EP11	EP02	0.4018
CK	EP28	EP02	0.0278
CK	EP25	EP02	0.2497
CK	EP33	EP02	0.3746
CK	EP09	EP02	0.2604
CK	EP14	EP02	0.8986
CK	EP35	EP02	0.8019
CK	EP34	EP02	0.4812
CK	EP11	EP21	0.0037
CK	EP28	EP21	0.6577
CK	EP25	EP21	0.9718
CK	EP33	EP21	0.2280
CK	EP09	EP21	0.6612
CK	EP14	EP21	0.0339
CK	EP35	EP21	0.7979
CK	EP34	EP21	1.0000
CK	EP02	EP21	0.6532
CK	EP11	EP20	0.4740
CK	EP28	EP20	0.7064
CK	EP25	EP20	0.9953
CK	EP33	EP20	0.7540
CK	EP09	EP20	0.0618
CK	EP14	EP20	0.7960
CK	EP35	EP20	0.7820
CK	EP34	EP20	0.1706
CK	EP02	EP20	0.9117
CK	EP21	EP20	0.6733

Plectropomus areolatus			
**Pop**	**Locus#1**	**Locus#2**	***P***
TS	Pb111	Pma038	0.7137
TS	Pb120	Pma038	0.0463
TS	Pa114	Pma038	0.8813
TS	Pa117	Pma038	0.3301
TS	Pm6	Pma038	0.6812
SO	Pm5	Pm3	0.1827
SO	Pm5	PaD2	0.9591
SO	Pm3	PaD2	0.7634
SO	Pm5	Pb111	0.6269
SO	Pm3	Pb111	0.5025
SO	PaD2	Pb111	0.5493
SO	Pm5	Pb120	0.1576
SO	Pm3	Pb120	0.0776
SO	PaD2	Pb120	0.3499
SO	Pb111	Pb120	0.8223
SO	Pm5	Pa114	0.2615
SO	Pm3	Pa114	0.5939
SO	PaD2	Pa114	0.6078
SO	Pb111	Pa114	0.0793
SO	Pb120	Pa114	0.2835
SO	Pm5	Pa117	0.8810
SO	Pm3	Pa117	0.3047
SO	PaD2	Pa117	0.8876
SO	Pb111	Pa117	0.5270
SO	Pb120	Pa117	0.7648
SO	Pa114	Pa117	0.7081
SO	Pm5	Pm6	0.3183
SO	Pm3	Pm6	0.5732
SO	PaD2	Pm6	0.9896
SO	Pb111	Pm6	0.7435
SO	Pb120	Pm6	1.0000
SO	Pa114	Pm6	0.6482
SO	Pa117	Pm6	0.1375
SO	Pm5	Pma038	0.9110
SO	Pm3	Pma038	0.0390
SO	PaD2	Pma038	0.0285
SO	Pb111	Pma038	0.2134
SO	Pb120	Pma038	0.8786
SO	Pa114	Pma038	0.2019
SO	Pa117	Pma038	0.3515
SO	Pm6	Pma038	0.1348
FJ	Pm5	Pm3	0.5820
FJ	Pm5	PaD2	0.0824
FJ	Pm3	PaD2	0.5621
FJ	Pm5	Pb111	0.3607
FJ	Pm3	Pb111	0.7029
FJ	PaD2	Pb111	0.3995
FJ	Pm5	Pb120	0.1477
FJ	Pm3	Pb120	0.9548
FJ	PaD2	Pb120	0.0081
FJ	Pb111	Pb120	0.5267
FJ	Pm5	Pa114	0.8538
FJ	Pm3	Pa114	0.9982
FJ	PaD2	Pa114	0.1730
FJ	Pb111	Pa114	0.2939
FJ	Pb120	Pa114	0.5513
FJ	Pm5	Pa117	0.9805
FJ	Pm3	Pa117	0.0429
FJ	PaD2	Pa117	0.7170
FJ	Pb111	Pa117	0.3774
FJ	Pb120	Pa117	0.5036
FJ	Pa114	Pa117	0.9401
FJ	Pm5	Pm6	1.0000
FJ	Pm3	Pm6	0.7962
FJ	PaD2	Pm6	0.6547
FJ	Pb111	Pm6	0.8325
FJ	Pb120	Pm6	0.8117
FJ	Pa114	Pm6	0.4756
FJ	Pa117	Pm6	0.9524
FJ	Pm5	Pma038	0.7875
FJ	Pm3	Pma038	0.8252
FJ	PaD2	Pma038	0.2413
FJ	Pb111	Pma038	0.7707
FJ	Pb120	Pma038	0.9910
FJ	Pa114	Pma038	0.8919
FJ	Pa117	Pma038	0.9809
FJ	Pm6	Pma038	0.6458

Plectropomus leopardus			
**Pop**	**Locus#1**	**Locus#2**	***P***
FJ	Ple002	Ple005	0.3665
FJ	Pma090	Ple005	0.0053
FJ	Pma112	Ple005	NA
FJ	Pm3	Ple005	1.0000
FJ	Pma022	Ple005	0.3328

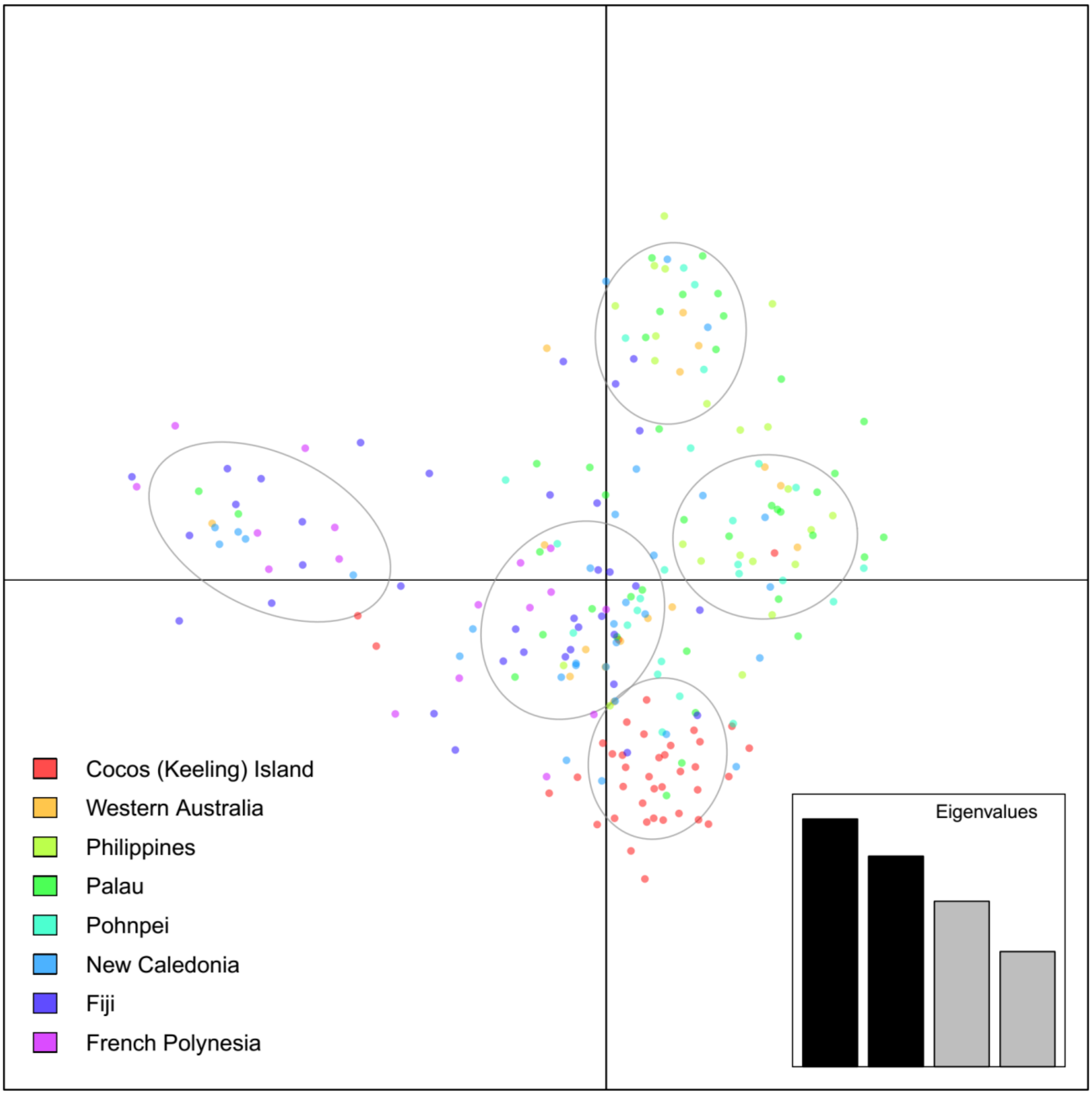

Figure S1. Discriminant analysis of principal components (DAPC) scatter plots of *Epinephelus polyphekadion* Central Indo-Pacific populations based on 11 microsatellite loci with 5 clusters defined.

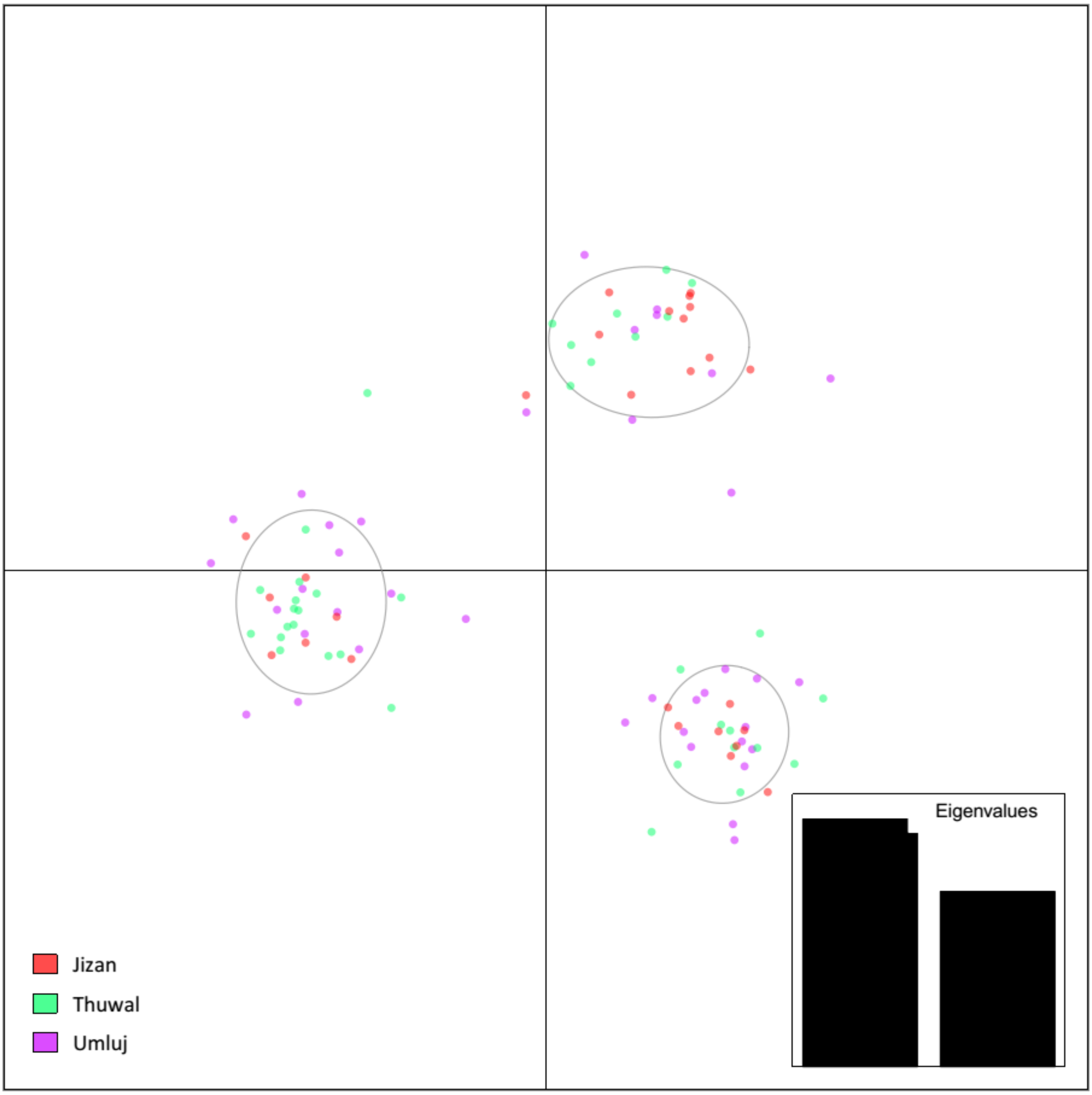

Figure S2. Discriminant analysis of principal components (DAPC) scatter plots of *Plectropomus areolatus* Red Sea populations based on 9 microsatellite loci with 3 clusters defined.

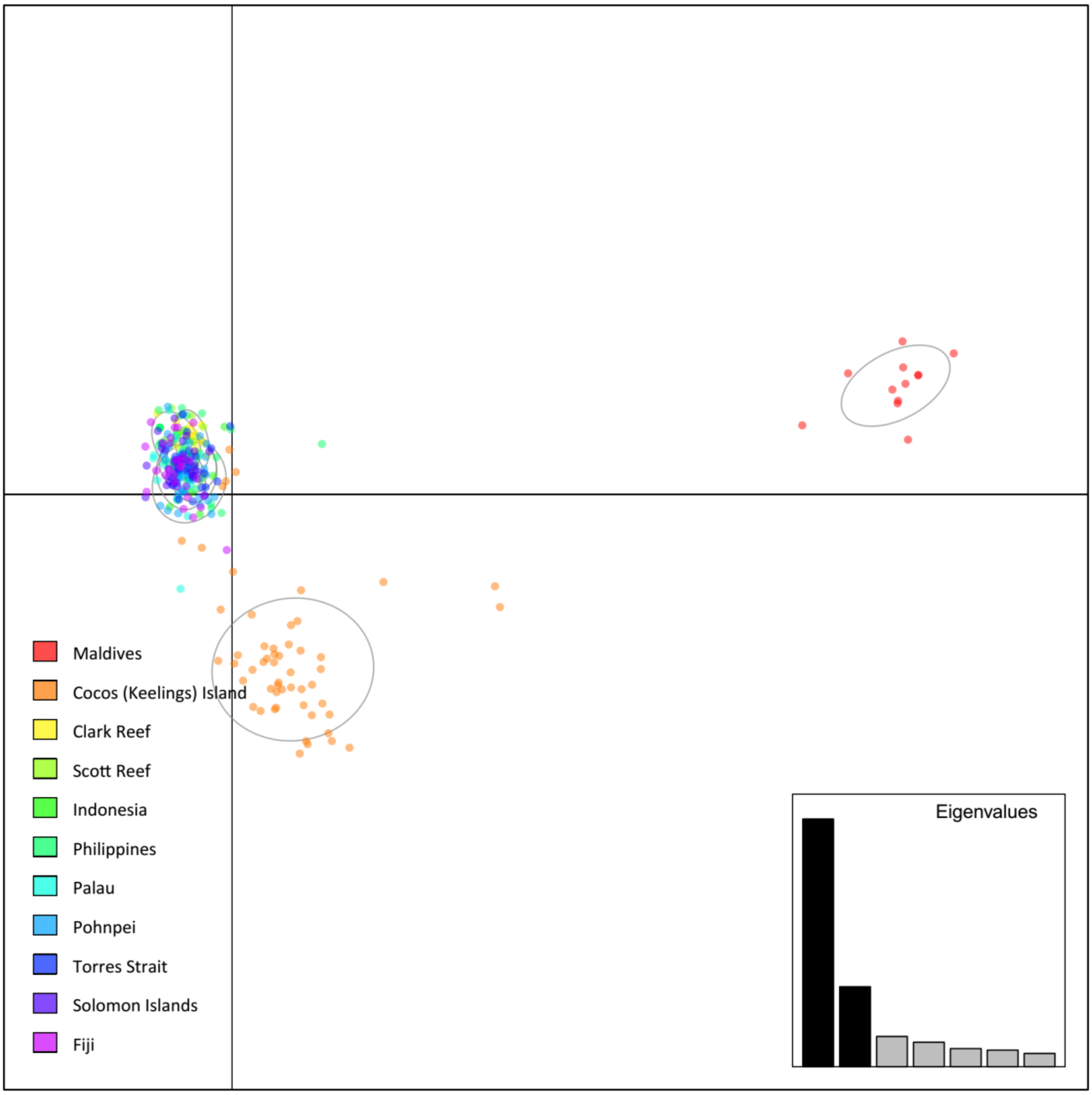

Figure S3. Discriminant analysis of principal components (DAPC) scatter plots of *Plectropomus areolatus* Central Indian Ocean Islands and Central Indo-Pacific populations based on 9 microsatellite loci with 9 clusters defined.

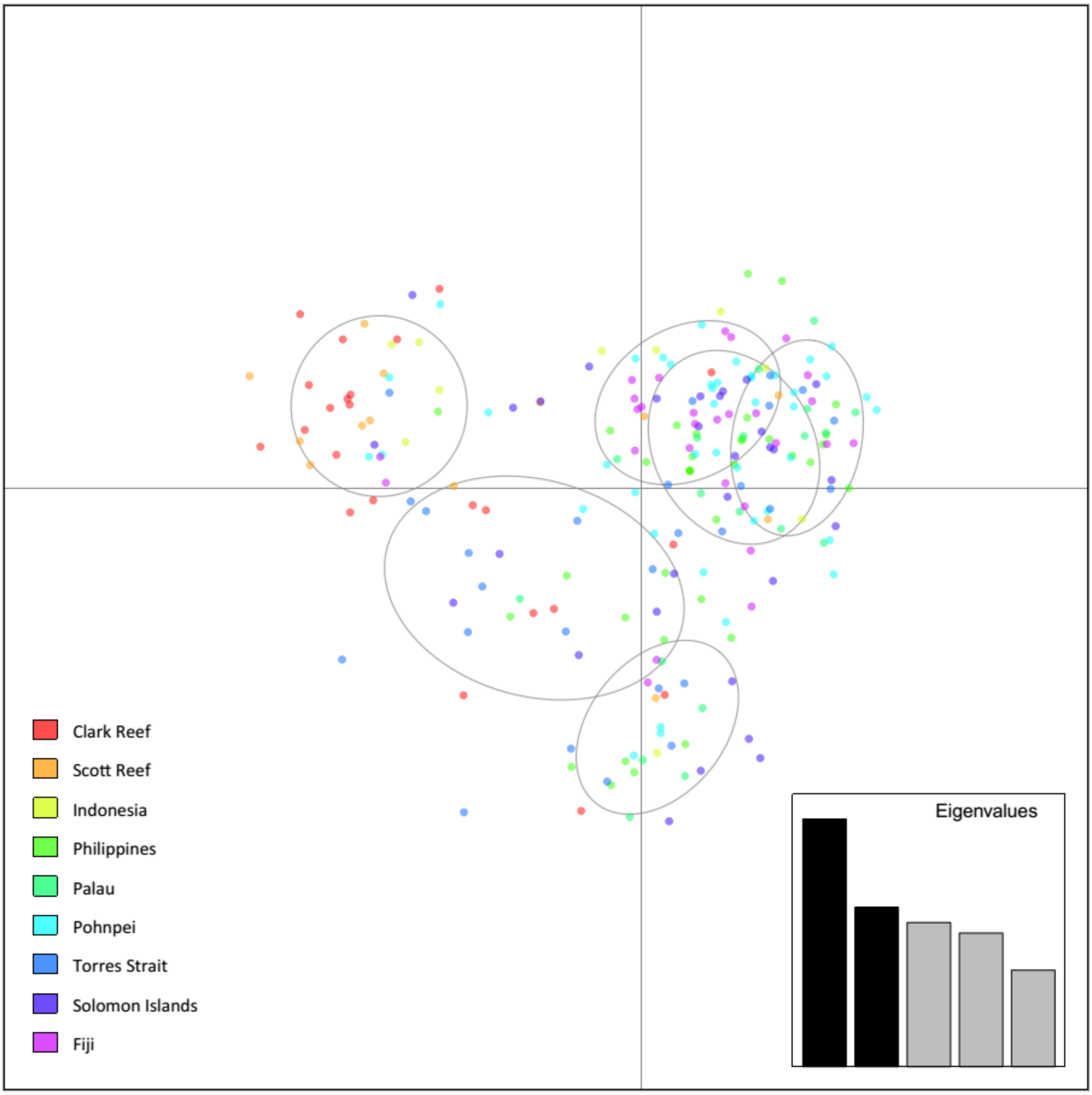

Figure S4. Discriminant analysis of principal components (DAPC) scatter plots of *Plectropomus areolatus* Western Australia + Pacific Ocean populations based on 9 microsatellite loci with 6 clusters defined.

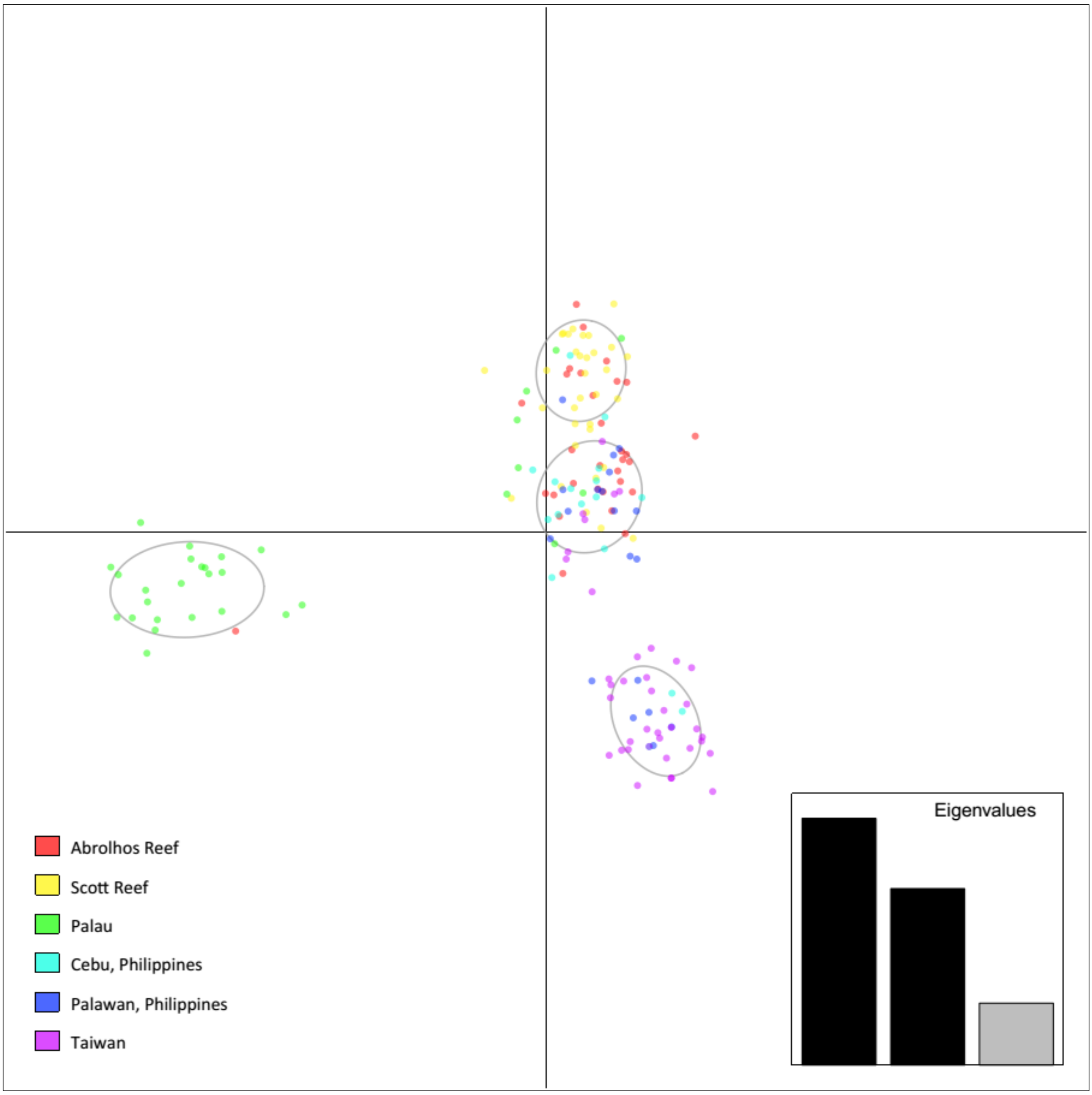

Figure S5. Discriminant analysis of principal components (DAPC) scatter plots of *Plectropomus leopardus* Western populations based on 9 microsatellite loci with 4 clusters defined.

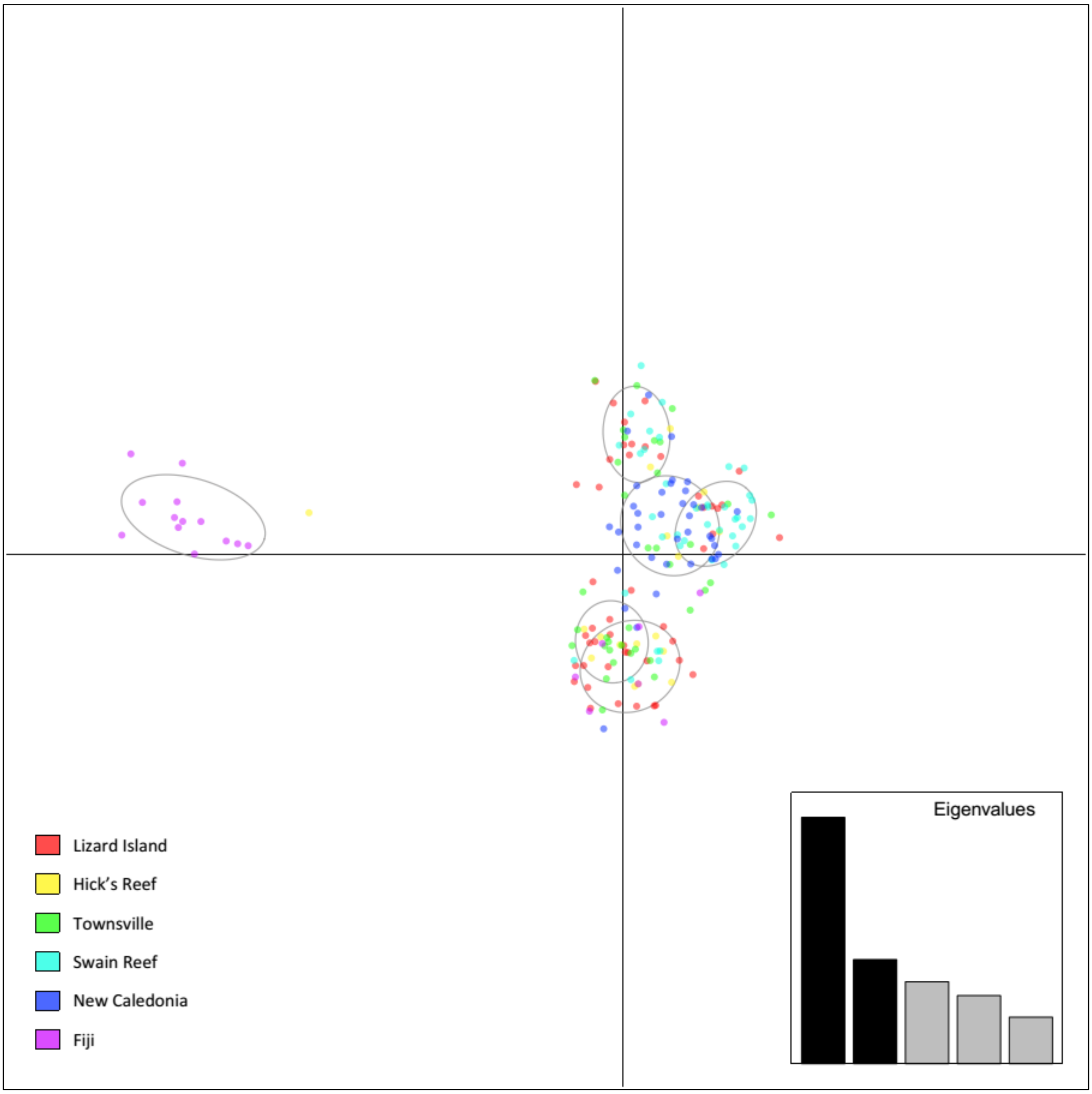

Figure S6. Discriminant analysis of principal components (DAPC) scatter plots of *Plectropomus leopardus* Eastern populations based on 9 microsatellite loci with 6 clusters defined.

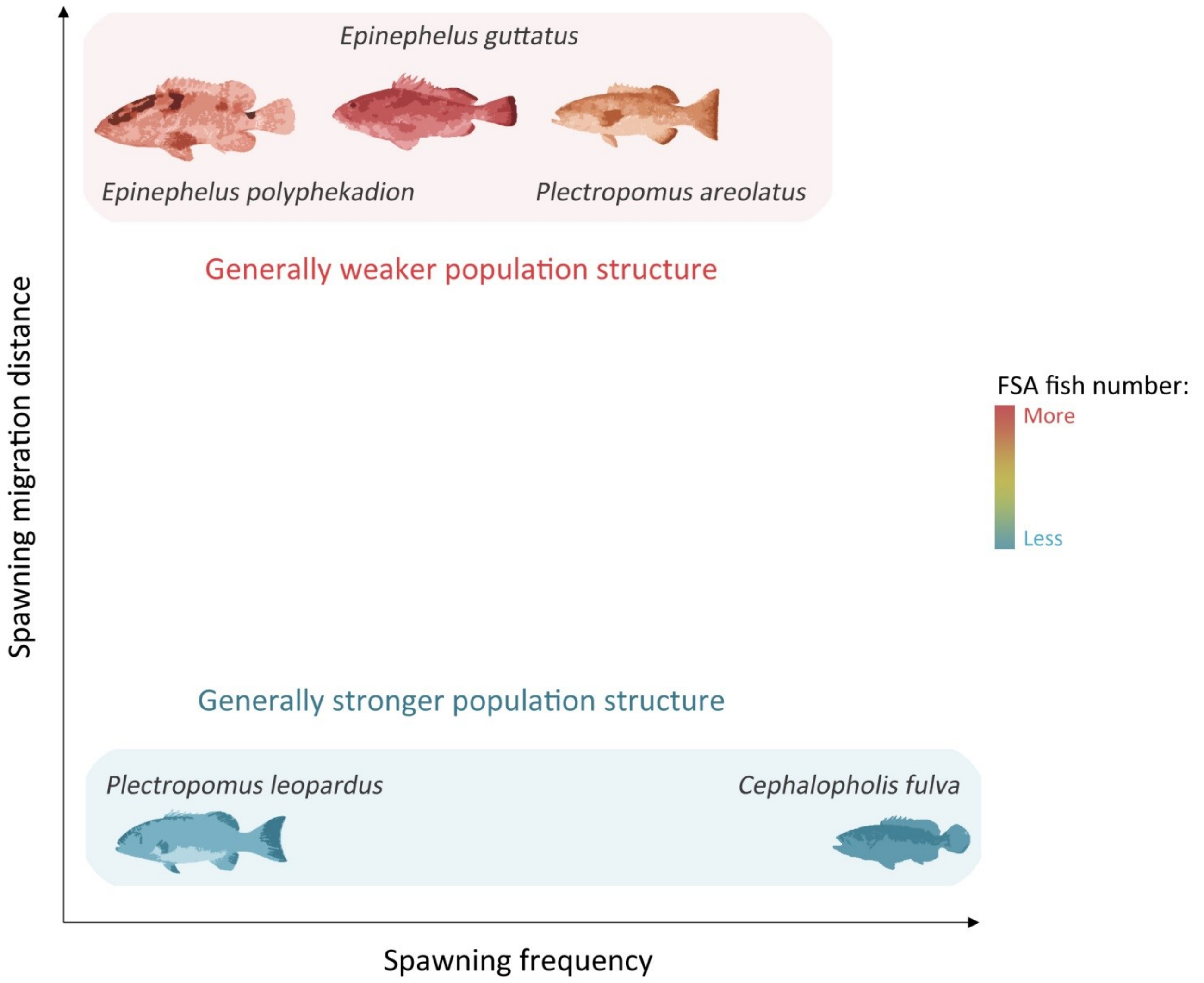

Figure S7. Summary of population genetic structure and reproductive characteristics of five grouper species analysed in this study and Portnoy et al. (2013).
